# Concerted Action of AMPK and Sirtuin-1 Induces Mitochondrial Fragmentation Upon Inhibition of Ca^2+^ Transfer to Mitochondria

**DOI:** 10.3389/fcell.2020.00378

**Published:** 2020-05-25

**Authors:** Alenka Lovy, Ulises Ahumada-Castro, Galdo Bustos, Paula Farias, Christian Gonzalez-Billault, Jordi Molgó, Cesar Cardenas

**Affiliations:** ^1^Department of Neuroscience, Center for Neuroscience Research, Tufts School of Medicine, Boston, MA, United States; ^2^Center for Integrative Biology, Faculty of Sciences, Universidad Mayor, Santiago, Chile; ^3^Geroscience Center for Brain Health and Metabolism, Santiago, Chile; ^4^Department of Biology, Faculty of Science, Universidad de Chile, Santiago, Chile; ^5^Université Paris-Saclay, CEA, Institut des Sciences du Vivant Frédéric Joliot, ERL CNRS n° 9004, Département Médicaments et Technologies pour la Santé, Service d’Ingénierie Moléculaire pour la Santé (SIMoS), Gif-sur-Yvette, France; ^6^The Buck Institute for Research on Aging, Novato, CA, United States; ^7^Department of Chemistry and Biochemistry, University of California, Santa Barbara, Santa Barbara, CA, United States

**Keywords:** IP3R channel, mitochondrial dynamics, actin, cortactin acetylation, Drp-1

## Abstract

Mitochondria are highly dynamic organelles constantly undergoing fusion and fission. Ca^2+^ regulates many aspects of mitochondrial physiology by modulating the activity of several mitochondrial proteins. We previously showed that inhibition of constitutive IP3R-mediated Ca^2+^ transfer to the mitochondria leads to a metabolic cellular stress and eventually cell death. Here, we show that the decline of mitochondrial function generated by a lack of Ca^2+^ transfer induces a DRP-1 independent mitochondrial fragmentation that at an early time is mediated by an increase in the NAD+/NADH ratio and activation of SIRT1. Subsequently, AMPK predominates and drives the fragmentation. SIRT1 activation leads to the deacetylation of cortactin, favoring actin polymerization, and mitochondrial fragmentation. Knockdown of cortactin or inhibition of actin polymerization prevents fragmentation. These data reveal SIRT1 as a new player in the regulation of mitochondrial fragmentation induced by metabolic/bioenergetic stress through regulating the actin cytoskeleton.

## Introduction

Mitochondria are a highly dynamic interconnected network of organelles that catalyze the synthesis of most cellular energy in the form of ATP, play a vital role in metal ion homeostasis, regulate several types of cell death and work as a hub for the biosynthesis of building blocks necessary for the generation of amino acids, lipids, and nucleotides (Ahn and Metallo, 2015; Spinelli and Haigis, 2018; Ward and Cloonan, 2019). Mitochondria also play a major role in the regulation of cellular calcium (Ca^2+^), shaping cytosolic Ca^2+^ signal by acting as a Ca^2+^ buffer to produce different cellular responses (Contreras et al., 2010). In addition to modulating the cytosolic Ca^2+^ patterns, the uptake of Ca^2+^ by mitochondria, mediated by the mitochondrial Ca^2+^ uniporter (MCU) (De Stefani et al., 2016), increases the activity of several proteins in the mitochondrial matrix including three key dehydrogenases of the tricarboxylic acid (TCA) cycle, the F1-F0 ATP synthase and the malate-aspartate shuttle (Glancy and Balaban, 2012; Del Arco et al., 2016). Ca^2+^ is mainly mobilized to the mitochondria from the endoplasmic reticulum (ER), the major Ca^2+^ storage organelle in most cells, with which it is functionally and physically coupled in special regions known as mitochondria-associated membranes (MAMs) (Csordas et al., 2010; Marchi et al., 2017). Ca^2+^ transfer from the ER to the mitochondria is mainly mediated by the inositol 1,4,5-trisphosphate (InsP3) receptors (IP3Rs), a family of three Ca^2+^ channels activated by InsP3 (Foskett et al., 2007). Physiological stimulation with InsP3-generating agonists generates an increase of cytosolic Ca^2+^ followed by a mitochondrial Ca^2+^ increase that induces a rise in the levels of NADPH due to a change in the pyruvate dehydrogenase (PDH) activity (Rizzuto et al., 1998; Robb-Gaspers et al., 1998). Corroborating this result, we found that in unstimulated conditions, the constitutive IP3R-mediated Ca^2+^ release is fundamental to maintain the optimal activity of PDH, energy levels and cellular homeostasis. In fact, inhibition of the Ca^2+^ transfer to mitochondria by inhibition of either the IP3R or MCU causes a bioenergetic crisis that activates AMPK and autophagy as a part of a survival response (Cardenas et al., 2010).

The energy status of the cell is often associated with specific mitochondrial morphologies, which are mainly the result of two highly dynamic processes such as the mitochondrial fusion and fission (Mishra and Chan, 2016). Increases in oxidative phosphorylation (OXPHOS) activity have been associated with fusion and elongation of the mitochondrial network in yeast (Egner et al., 2002; Jakobs et al., 2003) and mammalian cells (Rossignol et al., 2004; Mitra et al., 2009; Tondera et al., 2009). OXPHOS activity induces mitochondrial fusion by promoting the dimerization of mitofusin and organelle tethering (Shutt et al., 2012) and by stimulating the activity of the metalloprotease Yme1L which process Opa1, leading to activation of its fusion activity (Mishra et al., 2014). Conversely, OXPHOS inhibition and mitochondrial dysfunction induce fission and fragmentation of the mitochondrial network in several cellular contexts (Benard et al., 2007; Knott and Bossy-Wetzel, 2008; Liot et al., 2009). Nevertheless, mitochondrial fission plays a fundamental physiological role in mitochondrial transport, mitophagy and apoptosis (Suen et al., 2008; Mao and Klionsky, 2013; Fukumitsu et al., 2016; Burman et al., 2017; Trevisan et al., 2018; Wang and Zhang, 2018). Mitochondrial fission is a multi-step process that in most scenarios, depends on the dynamin-related protein1 (Drp1), which upon stimulation is recruited to the outer mitochondrial membrane where it forms a multimeric ring-like structure at mitochondrial fission sites that constricts and subsequently splits mitochondria (Kraus and Ryan, 2017). Mitochondrial fission also involves dynamin-2 (Dnm2) (Lee et al., 2016), the endoplasmic reticulum (Friedman et al., 2011), myosin II (Korobova et al., 2014), actin (Li et al., 2015) and actin associated proteins such as INF2 (Korobova et al., 2013), Spire 1C (Manor et al., 2015), and cofilin (Li et al., 2018). Mitochondrial fission occurs at the mitochondria–ER contacts, where the ER initiates the process by surrounding mitochondria and marking the constriction site (Friedman et al., 2011). Then, the ER-bound INF2 and the mitochondrial Spire1C induces actin nucleation and polymerization at this site (Manor et al., 2015), which most likely, provides the mechanical force to drive mitochondrial pre-constriction together with Myosin IIa (Korobova et al., 2014). At this site, MFF and MiDs recruit Drp1 where it oligomerizes to a ring-like structure enhancing the pre-existing mitochondrial constriction site in a GTP-hydrolysis fashion (Loson et al., 2013). Finally, Dnm2 is recruited to Drp1-mediated mitochondrial constriction neck where it assembles and terminates membrane scission (Lee et al., 2016). The actin-binding proteins cofilin and cortactin are also involved in this process (Li et al., 2015, 2018; Rehklau et al., 2017), but their exact role is less clear. Nonetheless, Drp1-independent mitochondrial fission has been described (Pagliuso et al., 2018). AMPK, the main energetic sensor of the cell (Hardie et al., 2016), regulates various aspects of the mitochondrial biology including mitochondrial biogenesis, mitochondrial quality control, and mitochondrial dynamics (Herzig and Shaw, 2018). Regarding AMPK, it has been known for a long time that suppression or reduction of mitochondrial ATP synthesis triggers mitochondrial fragmentation and the mechanism behind this phenomenon was recently unveiled by Toyama et al. (2016) who show that under bioenergetic stress induced by OXPHOS inhibitors, activated AMPK in turn activates the Drp1 receptor Mff by phosphorylation, promoting the recruitment of Drp1 to constriction sites. In addition to AMPK, sirtuins, a family (SIRT1–SIRT7) of evolutionarily conserved NAD+-dependent deacetylases, have emerged as crucial sensors of metabolic and cellular energy balance (Giblin et al., 2014). SIRT3, SIRT4, and SIRT5, reside within the mitochondria, where they modify subunits of the electron transport chain (ETC), enzymes of the TCA cycle (Hebert et al., 2013; Park et al., 2013) and enzymes involved in fatty acid oxidation, and amino acid metabolism (Haigis et al., 2006; Csibi et al., 2013; Jeong et al., 2013). Moreover, SIRT3 and SIRT5 participate in the regulation of mitochondrial dynamics, as their activation protects from fragmentation (Morigi et al., 2015; Guedouari et al., 2017; Pillai et al., 2017). Interestingly, the activation of SIRT1 by increases in the NAD+ levels or by resveratrol, induces mitochondrial fragmentation (Ren et al., 2017), suggesting the presence of a specific mechanism that regulates mitochondrial dynamics inside and outside the mitochondria. Here we show that the bioenergetic/metabolic stress caused by lack of Ca^2+^ transfer from the ER to the mitochondria induces mitochondrial fragmentation through a mechanism mediated by the action of SIRT1 on cortactin at an early time point, which is followed by the activation of AMPK acting through Drp1 and SIRT1 upon a sustained bioenergetic/metabolic stress.

## Results

### The Lack of Ca^2+^ Transfer to Mitochondria Induces Mitochondrial Fragmentation

**FIGURE 1:**
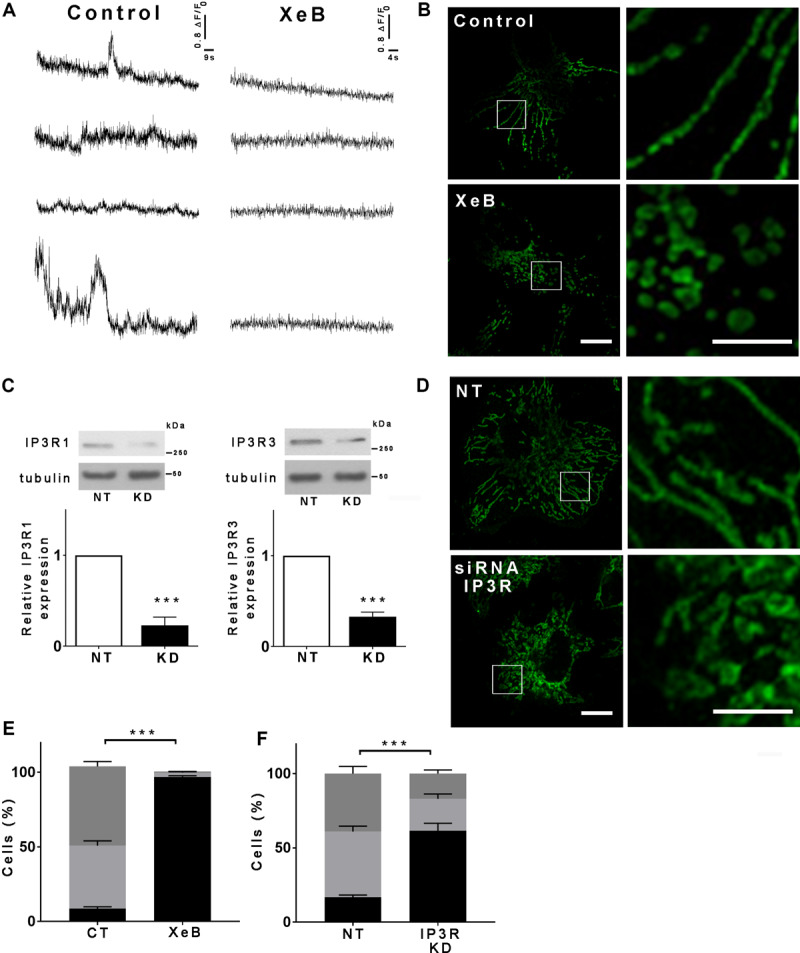
Inhibition of IP3R-mediated calcium transfer to mitochondria causes mitochondrial fragmentation. **(A)** Representative recordings of spontaneous Ca^2+^ release events observed by TIRF microscopy in unstimulated Hela cells treated or not with 5 μM XeB. Every trace represents a Fluo-4-AM fluorescence ratio (ΔF/F_0_) where ΔF corresponds to the fluorescence change at each pixel relative to the mean resting fluorescence (F0) before stimulation. **(B)** Hela cells were treated with 5 μM XeB or vehicle for 60 min and then immunostained with anti-TOMM20 to detect mitochondria. Bar: 10 μm. **(C)** Representative Western blots of type 1 and 3 IP3R in Hela cell simultaneously transfected with siRNA against type 1 and type 3 IP3Rs (KD) or a non-target (NT) siRNA as control. Bar graph: IP3R1/tubulin and IP3R3/tubulin expressed as average fold increase over basal levels (control cells, NT). Mean ± SEM of 3 independent experiments with 5 replicates each. ***P < 0.001 compared to control. **(D)** Confocal images of Hela cells simultaneously transfected with siRNA for type 1 and type 3 IP3Rs (KD) or a non-target (NT) siRNA as control for 48 h and immunostained with anti-TOMM20 to detect the mitochondrial network. Bar: 10 μm. **(E,F)** Mitochondrial morphology analysis of cells treated with XeB **(B)** or knockdown (KD) for IP3Rs **(D)**. Fragmented (black), ≤1 μm, medium (light gray), ≥1 and ≤4 μm, networked (dark gray), ≥4 μm. Data represent mean ± SEM of 3 independent experiments. For the analysis of the mitochondrial network 150 cells/condition were scored in each of the 3 independent experiments ***p < 0.001.

**FIGURE 2:**
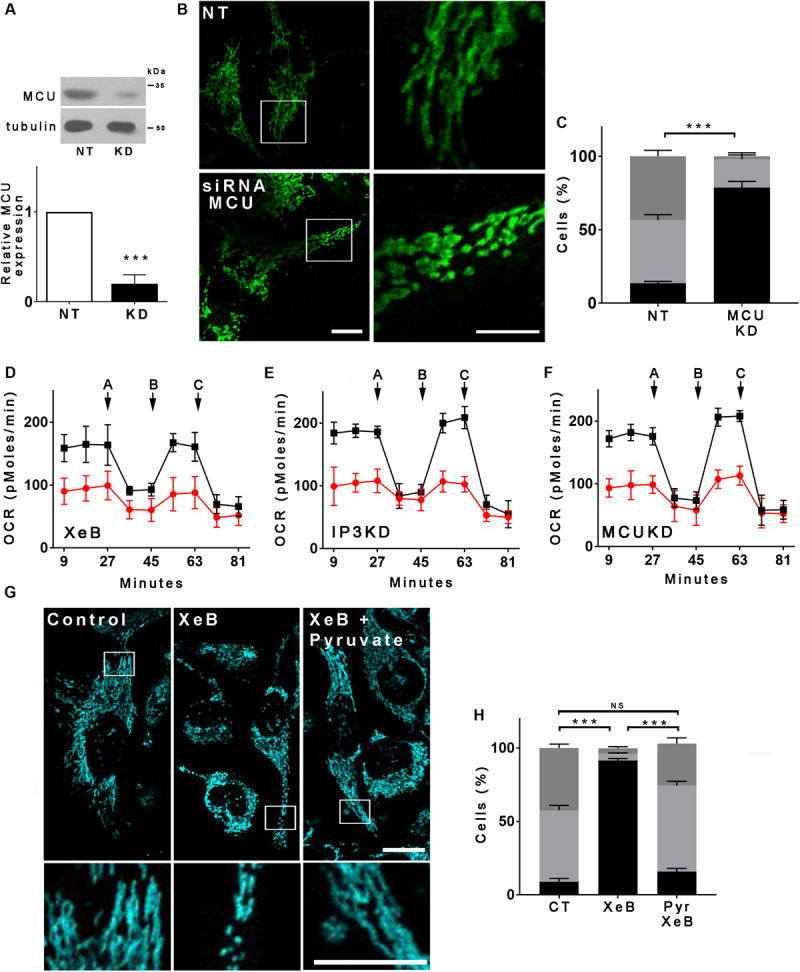
The bioenergetic stress caused by the inhibition of IP3R-mediated Ca^2+^ transfer to the mitochondria is responsible for the mitochondrial network fragmentation. **(A)** Representative Western blot of MCU in HeLa cells transfected with a siRNA against MCU (KD) or a non-target (NT) siRNA as control. Bar graph: MCU/tubulin expressed as average fold increase over basal levels (control cells, NT). Mean ± SEM of 3 independent experiments with 4 replicates each. ***P < 0.001 compared to control. **(B)** Confocal images of Hela cells transfected with siRNA for MCU (KD) or a non-target (NT) siRNA as control for 72 h and immunostained with anti-TOMM20 to detect the mitochondrial network. Bar: 10 μm. **(C)** Mitochondrial morphology analysis of MCU knockdown (KD) cells on **(B)**; Fragmented (black), ≤1 μm, medium (light gray), ≥1 and ≤4 μm, networked (dark gray), ≥4 μm. **(D–F)** Basal and maximum oxygen consumption rate (OCR) determined by Seahorse after the addition of oligomycin (a), FCCP (b), and rotenone (c) in HeLa cells treated with XeB or knockdown (KD) for IP3Rs or MCU. **(G)** Confocal images of Hela cells treated with 5 μM XeB for 60 min in the presence of methyl-pyruvate immunostained with anti-TOMM20 to detect the mitochondrial network. Bar: 10 μm. **(H)** Mitochondrial morphology analysis of MCU knockdown (KD) cells on **(B)**; Fragmented (black), ≤1 μm, medium (light gray), ≥1 and ≤4 μm, and networked (dark gray), ≥4 μm. Data represent mean ± SEM of 3 independent experiments. In each experiment 150 cells/condition were scored. ***p < 0.001. ns, not significant.

Spontaneous low-level IP3R-mediated Ca^2+^ transfer to mitochondria is essential for optimal oxidative phosphorylation (OXPHOS) and ATP production. Inhibition of IP3R activity or mitochondrial Ca^2+^ uptake decreases pyruvate dehydrogenase activity, generating a bioenergetic crisis characterized by reduced oxygen consumption, AMPK, and autophagy activation in several cell types (Cardenas et al., 2010, 2016). As mitochondrial function is related to its morphology, we inhibited spontaneous IP3R-mediated Ca^2+^ signals in HeLa cells with the specific IP3R inhibitor Xestospongin B (XeB, 5 μM) for 1 h (Figure 1A) and mitochondrial morphology was determined by immunolabeling of the outer mitochondrial membrane (OMM) protein TOMM20 with confocal microscopy. As shown in Figures 1B,E, control cells reveal an interconnected network of long tubular mitochondria, which almost completely fragments into round-shaped mitochondria upon inhibition of IP3R. Transmission electron microscopy (TEM) confirmed the presence of numerous small rounded mitochondria in the XeB treated cells, while control cells show three to four-times longer mitochondria (Supplementary Figure S1A). Notably, mitochondrial fragmentation was not accompanied by evident structural rearrangements of the cristae (Supplementary Figure S1B). The fragmentation induced by IP3R inhibition with XeB was also observed in cell lines derived from glioma (U-87), breast (MCF10A), kidney (HEK293), and primary human fibroblasts (HF) (Supplementary Figure S2). To discard that the mitochondrial fragmentation observed corresponds to an off-target effect of XeB, type 1, and type 3 IP3R were simultaneously knocked down with a pool of siRNA [69 ± 3 and 61 ± 3.6%, respectively] (Figure 1C) and the status of the mitochondrial network determined. As shown in Figures 1D,F, the reduction of IP3R expression also induced mitochondrial fragmentation in a significant part of the cell population. Moreover, the incubation of HeLa cells with the permeable chelator BAPTA-AM (1 μM, 1 h), but not the pH sensor BCECF-AM (1 μM, 1 h), had a similar effect (Supplementary Figures S3A,B) confirming the essential role of Ca^2+^ signal to maintain the mitochondrial network. Mitochondrial fragmentation is usually accompanied by loss of mitochondrial membrane potential (ΔΨm). Thus, to determine the effect of IP3R inhibition on ΔΨm we labeled the cells with TMRE in non-quenching mode (8 nM TMRE for 1 h), treated with 5 μM XeB and imaged every 15 min. As shown in the representative confocal images of Supplementary Figure S3C, the inhibition of IP3R induces mitochondrial hyperpolarization rather than a loss of ΔΨm, a result confirmed by flow cytometry (Supplementary Figure S3D). Autophagy machinery is capable of inducing mitochondrial fission (Yamashita et al., 2016) and since the inhibition of Ca^2+^ transfer to the mitochondria induces autophagy (Cardenas et al., 2010) we determined whether the autophagosomes colocalized with mitochondria. As shown in Supplementary Figure S3E, despite a great increase in autophagosome formation, not much colocalization with mitochondria is observed. To determine whether the lack of Ca^2+^ transfer from the endoplasmic reticulum (ER) to mitochondria is responsible for the mitochondrial fragmentation and no other cellular mechanism dependent on IP3R-mediated Ca^2+^ signals, a transient knockdown of the mitochondrial Ca^2+^ uniporter (MCU), the protein responsible of the uptake of Ca^2+^ in the mitochondria (De Stefani et al., 2011) was performed with a specific pool of siRNA and mitochondrial morphology was evaluated. As shown in Figure 2A, 90% MCU knockdown (Figure 2A) caused a dramatic fragmentation of the mitochondrial network compared with control cells treated with a non-target siRNA (Figures 2B,C). These results suggest that Ca^2+^ transfer from the ER to mitochondria is essential to maintain the mitochondrial network. The interruption of spontaneous Ca^2+^ transfer from the ER through the IP3R to mitochondria causes a decrease in mitochondrial function as determined by oxygen consumption rate (OCR) after either inhibition with XeB (5 μM, 1 and 4 h) or siRNA-induced knockdown of the IP3Rs (24 h) or MCU (72 h) (Figures 2D,F). Thus, to determine whether the bioenergetic crisis generated by the lack of mitochondrial function or an independent phenomenon is what causes the disruption in the mitochondrial network, we inhibited the IP3R with XeB (5 μM, 4 h) in the presence of the mitochondrial substrate methyl-pyruvate (5 mM), which energizes mitochondria overcoming the bioenergetic effects induced by the ER-mitochondrial Ca^2+^ transfer inhibition (Cardenas et al., 2010, 2016). Surprisingly, methyl-pyruvate was able to protect the mitochondrial network from fragmentation (Figures 2G,H), confirming that it is the mitochondrial reduced-function that causes the loss of mitochondrial network integrity.

All together our results demonstrate that lack of Ca^2+^ transfer to mitochondria reduces its activity causing mitochondrial fragmentation.

### Mitochondrial Reduced-Function Induces Fragmentation and Is Mediated by SIRT1

**FIGURE 3:**
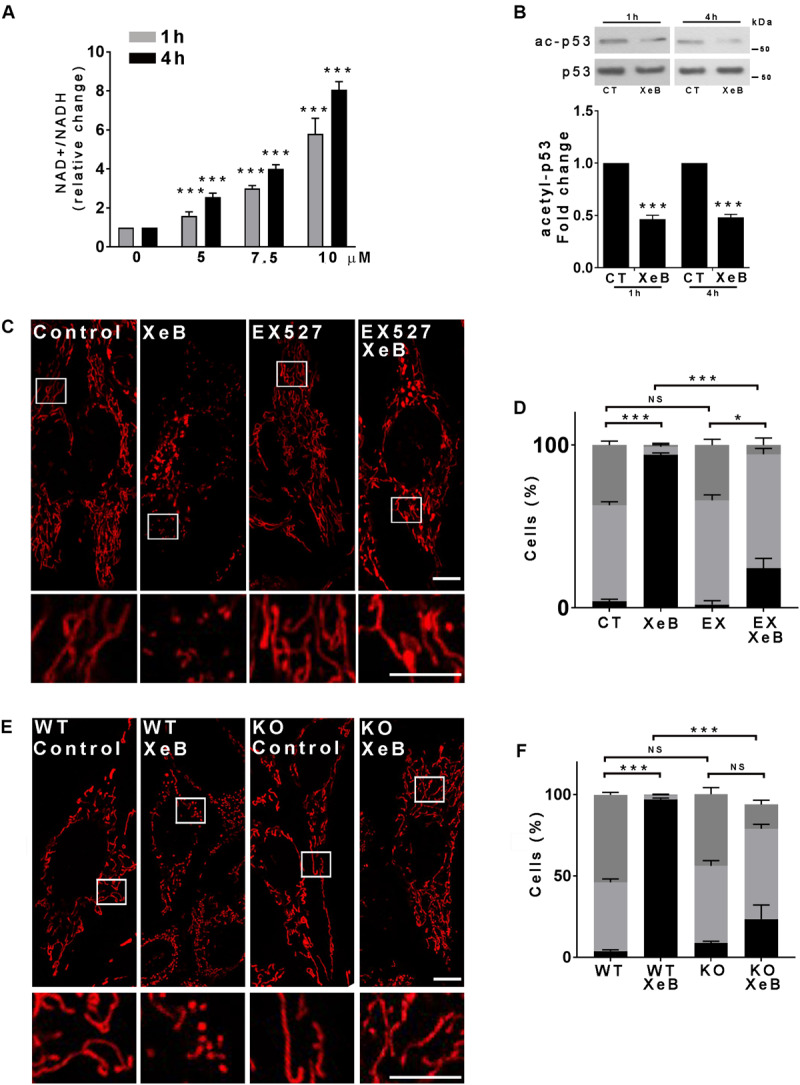
The activation of SIRT1 is necessary for the mitochondrial fragmentation induced by inhibition of IP3R-mediated Ca^2+^ transfer to the mitochondria. **(A)** Inhibition of IP3R with increasing concentrations of XeB for 1 and 4 h increases the NAD/NADH ratio in HeLa cells. **(B)** Representative Western blots of acetylated p53 (ac-p53) and p53 as loading control after inhibition of IP3R with 5 μM XeB for 1 and 4 h. Bar graph: ac-p53/p53 expressed as average fold increase over basal levels (control cells, CT). Mean ± SEM of 4 independent experiments with 3 replicates each. ***P < 0.001 compared to control. **(C)** Confocal images of Hela cells labeled with 8 nM TMRE to visualize mitochondria simultaneously treated with 5 μM XeB for 4 h and the SIRT1 inhibitor EX527 (12.5 μM). Bar: 10 μm. **(D)**. Mitochondrial morphology analysis of HeLa cells simultaneously treated with 5 μM XeB for 4 h and the SIRT1 inhibitor EX527 (12.5 μM) (C); Fragmented (black), ≤1 μm, medium (light gray), ≥1 and ≤4 μm, networked (dark gray), ≥4 μm. Data represent mean ± SEM of 3 independent experiments. In each experiment 150 cells/condition were scored. ***p < 0.001. ns, not significant. **(E)** Confocal images of SIRT1 knockout (KO) MEF cells labeled with 8 nM TMRE to visualize mitochondria treated with 5 μM XeB for 4 h. Bar: 10 μm. **(F)** Mitochondrial morphology analysis of SIRT1 KO MEF cells treated with 5 μM XeB for 4 h **(E)**; Fragmented (black), ≤1 μm, medium (light gray), ≥1 and ≤4 μm, networked (dark gray), ≥4 μm. Data represent mean ± SEM of 3 independent experiments. In each experiment 150 cells/condition were scored. ***p < 0.001. ns, not significant.

The acute inhibition of IP3R-mediated Ca^2+^ transfer to the mitochondria lowers cell ATP levels and activates AMPK (Supplementary Figures S4A,B) as described before (Cardenas et al., 2010, 2016), which has recently been shown to participate in the mitochondrial fragmentation induced by energy stress (Toyama et al., 2016). To determine whether AMPK plays a role in mitochondrial fragmentation we inhibited IP3R with XeB in the presence of compound C (CC), a known AMPK inhibitor, for 4 h and mitochondrial morphology was determined. Unexpectedly, the inhibition of AMPK was unable to prevent mitochondrial fragmentation (Supplementary Figures S4C,D). Another important cellular bioenergetic sensor is the NAD+ -dependent deacetylase SIRT1 (Chang and Guarente, 2014). Since the inhibition of IP3R affects the activity of the TCA cycle enzyme pyruvate dehydrogenase and possibly the activity of the isocitrate and the α-ketoglutarate dehydrogenases, which are Ca^2+^ dependent (Glancy and Balaban, 2012), it is expected that in addition to the drop in the ATP levels, the NAD^+^/NADH ratio also changes, therefore affecting the activity of SIRT1. We therefore determined the NAD^+^/NADH ratio after IP3R inhibition, finding a significant increase that was time and concentration dependent (Figure 3A). As an increase in the NAD^+^/NADH ratio likely activates SIRT1, we determined the levels of acetylation of p53, a known substrate of SIRT1 by Western blot, finding a significant decrease after 1 h or 4 h treatment with the IP3R inhibitor XeB (Figure 3B). Interestingly, the expression levels of SIRT1 significantly increased after 4 h of IP3R inhibition with XeB (Supplementary Figure S5A). To determine if SIRT1 participates in the mitochondrial fragmentation induced by the bioenergetic stress caused by IP3R inhibition, we simultaneously inhibited IP3R with 5 μM XeB and SIRT1 activity with 12.5 μM EX527 and mitochondrial morphology was determined after 4 h. As shown in Figures 3C,D, the inhibition of SIRT1 protected the mitochondrial network from fragmentation. To further confirm the role of SIRT1 in mitochondrial fragmentation we evaluated the status of the mitochondrial network in SIRT1 knockout (KO) MEF cells. A robust mitochondrial network was observed in both wild-type (WT) and SIRT1 KO MEF cells in basal conditions, however, upon IP3R inhibition with XeB (5 μM, 4 h) the mitochondrial network completely fragmented in control cells, while remaining unchanged in SIRT1 KO cells (Figures 3E,F). To determine whether the activation of SIRT1 was sufficient to induce mitochondrial fragmentation, we treated Hela cells with β-nicotinamide mononucleotide (NMN), a molecule known to increase the levels of NAD+ and activate SIRT1 (Imai and Guarente, 2016) for 12 h. As is shown in Supplementary Figures S5B,C, NMN treatment induced fragmentation of the mitochondrial network. Altogether, our data show that the mitochondrial fragmentation generated by the bioenergetic crisis induced by inhibition of Ca^2+^ transfer to the mitochondria is mediated by SIRT1.

### Mitochondrial Reduced-Function Induced Fragmentation Is Partially Drp1 Independent

**FIGURE 4:**
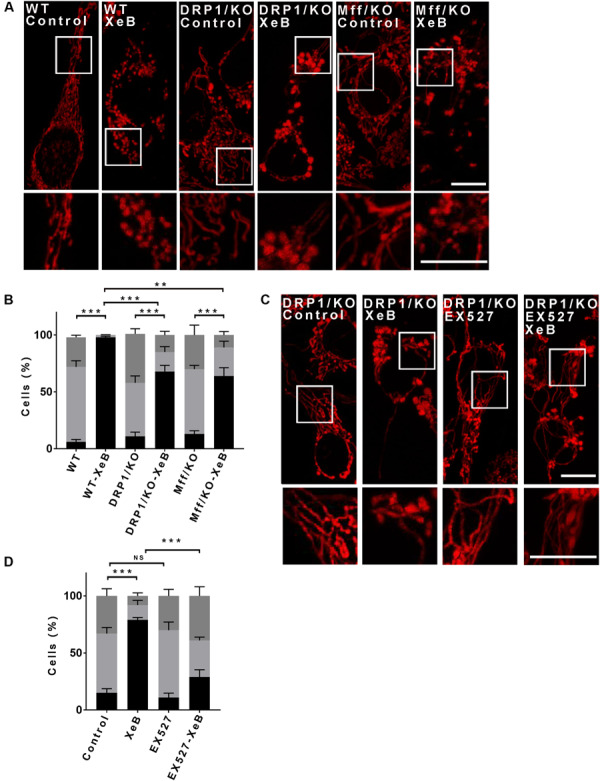
The mitochondrial fragmentation induced by inhibition of Ca^2+^ transfer to the mitochondria proceeds independently of Drp1. **(A)** Representative confocal images of HCT116 cells knockout (KO) for Drp1 and Mff labeled with 8 nM TMRE to visualize mitochondria treated with 5 μM XeB for 4 h Bar: 10 μm. **(B)** Mitochondrial morphology analysis of HCT116 cells KO for Drp1 and Mff treated with 5 μM XeB for 4 h **(A)**; fragmented (black), ≤1 μm, medium (light gray), ≥1 and ≤4 μm, networked (dark gray), ≥4 μm. Data represent mean ± SEM of 3 independent experiments. In each experiment 150 cells/condition were score. **p < 0.01, ***p < 0.001. ns, not significant. **(C)** Representative confocal images of HCT116 cells KO for Drp1 simultaneously treated with 5 μM XeB for 4 h and the SIRT1 inhibitor EX527 (12.5 μM). Bar: 10 μm. **(D)** Mitochondrial morphology analysis of HCT116 cells KO for Drp1 simultaneously treated with 5 μM XeB for 4 h and the SIRT1 inhibitor EX527 (12.5 μM) **(C)**; Fragmented (black), ≤1 μm, medium (light gray), ≥1 and ≤4 μm, networked (dark gray), ≥4 μm. Data represent mean ± SEM of 3 independent experiments. In each experiment 150 cells/condition were scored. **p < 0.01, ***p < 0.001, ns, not significant.

Mitochondrial fragmentation in most scenarios is mediated by the dynamin-related protein1 (Drp1), which upon mitochondrial fission stimulation, is recruited to specific mitochondrial membrane sites in the OMM, where it interacts with several receptors to mediate mitochondrial fission (Kraus and Ryan, 2017). We expressed a mCherry-Drp1 (Friedman et al., 2011) in Hela cells to determine whether its mitochondrial localization increases after IP3R inhibition with XeB. As shown in Supplementary Figure S6A, the mitochondrial network fragmented after 4 h of treatment with XeB without an increase in Drp1 localization with mitochondria. This data suggest that Drp1 may not be involved in the mitochondrial fragmentation observed after IP3R inhibition. To prove this hypothesis, Drp1- KO and WT HCT116 cells were treated with the IP3R inhibitor XeB (5 μM) for 4 h, and the mitochondrial network status was determined (Figures 4A,B). In WT HCT116 cells we observed a total fragmentation of the mitochondrial network after the treatment. Surprisingly, in Drp1- KO HCT116 cells, a significant number of fragmented mitochondria (68 ± 5%) was observed. A similar result was observed when we expressed a dominant-negative (72 ± 4%) mutant form of Drp1 (K38A) (Smirnova et al., 1998; Supplementary Figures S6B–D). To ensure that the Drp1-independent fragmentation was not the result of a compensatory mechanism induced by the absence of Drp1 activity, we proceeded to inhibit the IP3R in HCT116 cell lacking the Drp1 receptor Mff (Mff-KO), and the same results were obtained; fragmentation of most of the mitochondrial network (64 ± 7%) (Figures 4A,B). To determine whether SIRT1 is responsible for the fragmented mitochondrial population observed in a context of Drp1-independent fragmentation, we simultaneously inhibited the IP3R with XeB and SIRT1 with EX527 in Drp1-KO cells. As shown in Figures 4C,D, the inhibition of SIRT1 significantly reduces the percentage of fragmented mitochondria with a concomitant increase in long mitochondria.

### Deacetylation of Cortactin and the Actin Cytoskeleton Mediate the Mitochondrial Fragmentation Induced by Inhibition of Ca^2+^ Transfer to the Mitochondria

**FIGURE 5:**
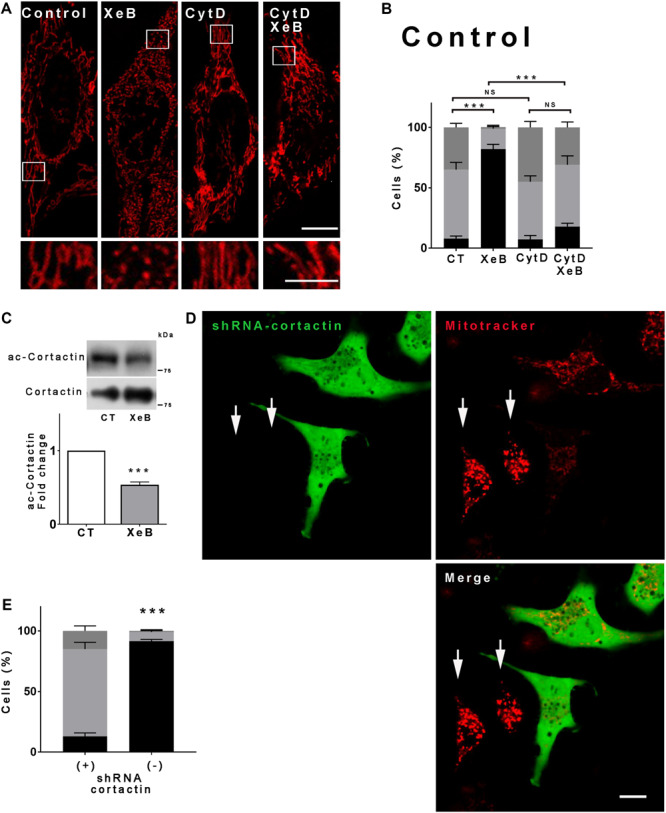
Deacetylation of cortactin and the polymerization of the actin cytoskeleton are required for the mitochondrial fragmentation induced by inhibition of the Ca^2+^ transfer to the mitochondria. **(A)** Representative confocal images of HeLa cells labeled with 8 nM TMRE to visualize mitochondria treated simultaneously with 5 μM XeB and 1 μM cytochalasin D for 4 h. Bar: 10 μm. **(B)** Mitochondrial morphology analysis of HeLa cells treated simultaneously with 5 μM XeB and 1 μM cytochalasin D for 4 h **(A)**; fragmented (black), ≤1 μm, medium (light gray), ≥1 and ≤4 μm, networked (dark gray), ≥4 μm. Data represent mean ± SEM of 3 independent experiments. In each experiment 150 cells/condition were scored. **p < 0.01, ***p < 0.001. ns, not significant. **(C)** Representative Western blot of acetylated cortactin (ac-cortactin) in HeLa cells treated with 5 μM XeB or vehicle for 4 h. Bar graph: ac-cortactin/cortactin expressed as average fold increase over basal levels (control cells, CT). Mean ± SEM of 3 independent experiments with 4 replicates each. ***P < 0.001 compared to control. **(D)** Representative confocal images of HeLa cells transfected with a vector engineered to simultaneously produce cortactin shRNA and GFP, loaded with mitotracker to visualize mitochondria and treated with 5 μM XeB for 4 h. Arrows point to untransfected cells. Bar: 10 μm. **(E)** Mitochondrial morphology analysis of HeLa cells transfected with cortactin shRNA and treated with 5 μM XeB for 4 h **(D)**; Fragmented (black), ≤1 μm, medium (light gray), ≥1 and ≤4 μm, networked (dark gray), ≥4 μm. Data represent mean ± SEM of 3 independent experiments. In each experiment 150 cells/condition were scored. **p < 0.01, ***p < 0.001. ns, not significant.

Next, we aimed to determine how SIRT1 promotes mitochondrial fragmentation. Actin and many of the proteins that regulate actin dynamics are subject to acetylation/deacetylation and several reports show that actin polymerization plays an important role in mitochondrial fragmentation (De Vos et al., 2005; DuBoff et al., 2012; Korobova et al., 2013; Stavru et al., 2013; Li et al., 2015). Thus, we inhibited the IP3R with XeB in presence or absence of cytochalasin D, which disrupts the actin cytoskeleton, and the mitochondrial morphology was determined. As shown in Figures 5A,B, disruption of the actin cytoskeleton prevented the mitochondrial fragmentation induced by IP3R inhibition. Similar results were observed when the actin cytoskeleton was disrupted with latrunculin B (Supplementary Figures S7A,B). These results suggest that actin polymerization is involved in the mitochondrial fragmentation. Cortactin is known to interact with filamentous actin (F-actin) and promotes actin polymerization (Weaver et al., 2001). Moreover, the acetylation of cortactin is known to impede its interaction with F-actin (Zhang et al., 2007) while its deacetylation increases actin polymerization (Ran et al., 2015). We determined the cortactin acetylation levels upon IP3R inhibition and observed a significant decrease of acetylation compared to the control (Figure 5C), which correlates with SIRT1 activation. To prove the involvement of cortactin in mitochondrial fragmentation we knocked down cortactin with a shRNA that concomitantly expresses a green fluorescence protein (GFP), which allowed us to discriminate the transfected from untransfected cells. As shown in Figures 5D,E and Supplementary Figure S7C, green transfected cells have a conserved mitochondrial network upon inhibition of IP3R with XeB while the untransfected cells fragment, confirming the role of cortactin.

### Upon Sustained Metabolic/Bioenergetic Stress Induced by Inhibition of the IP3R, Both AMPK and SIRT1 Participate in the Fragmentation of the Mitochondrial Network

**FIGURE 6:**
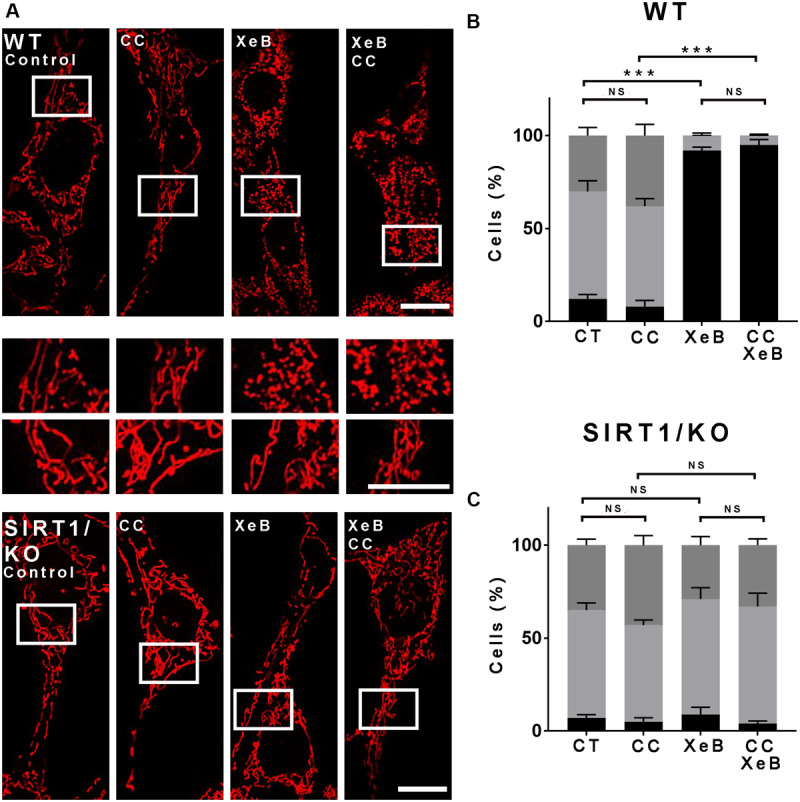
The mitochondrial fragmentation observed after inhibition of Ca^2+^ transfer to the mitochondria proceeds independently of AMPK at the earliest time points. **(A)** Representative confocal images of wild type (WT) and SIRT1 KO MEF cells labeled with 8 nM TMRE to visualize mitochondria treated simultaneously with 5 μM XeB and the AMPK inhibitor compound C (CC, 10 μM) for 4 h. Bar: 10 μm. **(B,C)** Mitochondrial morphology analysis of WT and SIRT1 KO MEF cells, respectively, treated simultaneously with 5 μM XeB and the AMPK inhibitor compound C (CC, 10 μM) for 4 h **(B)**; Fragmented (black), ≤1 μm, medium (light gray), ≥1 and ≤4 μm, networked (dark gray), ≥4 μm. Data represent mean ± SEM of 3 independent experiments. In each experiment 150 cells/condition were scored. **p < 0.01, ***p < 0.001. ns, not significant.

The lack of response to the AMPK inhibitor was puzzling, given the previous report that AMPK plays a significant role in inducing mitochondrial fragmentation upon bioenergetic stress (Toyama et al., 2016). Considering that this observation was obtained using AMPK KO cells, and to work under similar conditions, we decided to perform an overnight inhibition of both, the IP3R and AMPK. First, we determined the percentage of cell death induced by 5 μM XeB at different times, since we previously observed that the inhibition of IP3R with XeB induces cell death when cells enter mitosis, a phenomenon known as a mitotic catastrophe (Mc Gee, 2015). At 1, 4, or 8 h of XeB incubation, no cell death was observed (Supplementary Figure S8A). At 12 h, about 20% cell death was observed which increased by 24 h. Higher concentrations (7.5 and 10 μM) show a stronger effect increasing the amount of cell death at 12 h (Supplementary Figure S8B). We concluded that a 20% cell death would not interfere with our measurements and proceeded with the overnight experiments. Surprisingly, under these conditions, the inhibition of AMPK delivers a total protection of the mitochondrial network (Supplementary Figures S8C,D). In fact, the protection observed by an overnight AMPK inhibition seemed more profound that the one obtained by an overnight SIRT1 inhibition (Supplementary Figures S8C,D). These results show that mitochondrial fragmentation observed after the bioenergetic crisis generated by IP3R inhibition involves both AMPK and SIRT1 acting in a differently timed fashion. Given that AMPK inhibition delivers the major mitochondrial network protection after an overnight bioenergetic crisis, we hypothesized that in the long term, AMPK takes over and acts as an upstream regulator of SIRT1. If this is true, we would expect that inhibition of AMPK will not protect the mitochondrial network at early time points, but SIRT1 inhibition will. Therefore, we treated WT and SIRT1 knockout cells with XeB for 4 h in the presence of CC and observed the status of the mitochondrial network. As predicted, at the early time, CC did not protect the mitochondrial network from fragmentation (Figures 6A–C), suggesting that both AMPK and SIRT1 may be part of the same signaling pathway.

## Discussion

Mitochondria exist as a dynamic network that continuously fuse and divide in response to cellular needs, nutrient availability, and the metabolic state of the cell (Tilokani et al., 2018). Here, we show that the inhibition of constitutive IP3R-mediated Ca^2+^ transfer to mitochondria achieved either by pharmacological inhibition of the IP3R with Xestospongin B (XeB), the simultaneous knockdown of type 1 and type 3 IP3R or the knockdown of the MCU induces a bioenergetic/metabolic stress that triggers mitochondrial fragmentation. Ca^2+^ is essential for life and death and mitochondria play an active role in turning the balance to one side or the other (Westermann, 2010). In neurons, high K^+^ stimulation causes voltage-dependent Ca^2+^ channel (VDCC)-mediated Ca^2+^ entry that promotes mitochondrial fragmentation by halting mitochondria and activating Drp1 through Ca^2+^/calmodulin-dependent protein kinase (CaMKIα) phosphorylation (Han et al., 2008). In addition, a sustained increase in cytosolic Ca^2+^ caused by the activation of L-type Ca^2+^ channels, depolarization of the mitochondrial membrane potential (ΔΨm) or exposure to Ca^2+^ ionophores activate calcineurin, and trigger mitochondrial fragmentation through a mechanism that involves Drp1 dephosphorylation (Cribbs and Strack, 2007; Cereghetti et al., 2008). Nonetheless, loss of ΔΨm induces Drp1 dephosphorylation on serine 637 (Cereghetti et al., 2008), while activation of L-type Ca^2+^ channels induces dephosphorylation on serine 656 (Cribbs and Strack, 2007). Interestingly, all the stimuli mentioned above will increase mitochondrial Ca^2+^ uptake, which is necessary for mitochondrial fission as shown with a sustained Ca^2+^ increase induced by thapsigargin (Hom et al., 2007). In fact, recently it has been shown that mitochondrial Ca^2+^ influx initiates and sustains constriction of the mitochondrial inner compartment (CoMIC), which is necessary for mitochondrial fission (Cho et al., 2017). Moreover, the inverted formin 2 (INF2), which mediates actin polymerization, mitochondrial Drp1 recruitment and mitochondrial fission (Korobova et al., 2013), also favors the mitochondrial Ca^2+^ influx by enhancing the contact between the ER and mitochondria (Chakrabarti et al., 2018). Unexpectedly, here we show that the absence of Ca^2+^ transfer to the mitochondria also induces mitochondrial fragmentation. We previously demonstrated that IP3R mediated Ca^2+^ transfer to the mitochondria is essential to maintain the bioenergetic/metabolic homeostasis of normal and cancer cells (Cardenas et al., 2010, 2016). Inhibition of this signal results in inactivation of PDH, decrease in the levels of ATP, and activation of AMPK as a pro-survival response (Cardenas et al., 2010). AMPK activation induced by energetic stress caused by mitochondrial poisoning with rotenone and antimycin A induces mitochondrial fragmentation through a mechanism that involves phosphorylation of MFF and recruitment of Drp1 (Toyama et al., 2016). Here, despite an early activation of AMPK (Supplementary Figure S4) in response to the bioenergetic stress caused by IP3R inhibition, the mitochondrial fragmentation occurs independent of it, and is mainly driven by SIRT1. Importantly, the decrease in the TCA cycle activity caused by IP3R inhibition leads to an increase in the NAD+/NADH ratio that activates SIRT1 (Figure 3), while the inhibition of the electron transport chain (ETC) by rotenone, antimycin A, and metformin decrease rather than increase the NAD+/NADH ratio (Gui et al., 2016; Hou et al., 2018; Urra et al., 2018) preventing SIRT1 activation. Physiologically, SIRT1 and AMPK are concomitantly activated under conditions of reduced nutrient availability such as; glucose restriction, fasting, chronic calorie restriction, and exercise (Chen et al., 2008; Fulco et al., 2008; Canto and Auwerx, 2009; Canto et al., 2009; Costford et al., 2010). IP3R inhibition phenocopies a state of nutrient deprivation (Cardenas et al., 2010), and the activation of both AMPK and SIRT1 is expected. SIRT1 and AMPK can activate each other; AMPK can indirectly activate SIRT1 by elevating intracellular NAD+ levels (Fulco et al., 2008; Canto et al., 2009). On the other hand, SIRT1 has the ability to deacetylate and activate LKB1, which in turn activates AMPK (Hou et al., 2008; Lan et al., 2008). Here, we observed that prolonged (12 h) inhibition of AMPK delivers a more profound protection than a prolonged inhibition of SIRT1. It seems that AMPK may be acting up-stream of SIRT1 sustaining its activation directly (Lau et al., 2014) or by maintaining a high NAD+/NADH ratio (Canto et al., 2009). It is possible that without the action of AMPK, the compensatory rise in the glycolytic flux observed after IP3R inhibition (Cardenas et al., 2010) would recover the NADH (Kelly et al., 2018), which at this point could only be oxidized by the glycerol-3-phosphate shuttle. It is unlikely that it could be oxidized by the malate-aspartate shuttle in the mitochondria due to the need for Ca^2+^ (Lasorsa et al., 2003). Further experiments are necessary to fully understand the interplay between AMPK and SIRT1 in this context.

The increase in NAD+ induced by nicotinamide (NAM) has been associated with an increase in mitochondrial membrane potential, a feature also observed in our system, and a dramatic change in the mitochondrial structure from filaments to puncta or rings, that allows the clearance of mitochondria by mitophagy (Kang and Hwang, 2009). The inhibition of IP3R induced autophagy (Cardenas and Foskett, 2012) but no mitophagy was observed as determined by colocalization of LC3-GFP and mitotracker (data no shown). Later, the same group showed that the fragmentation induced by NAM required the presence of functional SIRT1 (Jang et al., 2012), however, no details were provided regarding the mechanism through which SIRT1 induced the fragmentation of mitochondria. In fact, no literature is available on this topic. Nevertheless, it is known that SIRT1 can modulate the actin cytoskeleton by deacetylating cortactin (Zhang et al., 2009), and the actin cytoskeleton has emerged as a major player in mitochondrial fragmentation (Hatch et al., 2014). One of the first observations came from Sheetz group who showed that pharmacological inhibition of F-actin polymerization reduces toxin-induced mitochondrial fragmentation (De Vos et al., 2005). A later work showed that treatment with actin polymerization inhibitor latrunculin B (LatB) induces mitochondrial elongation in U2OS cells (Korobova et al., 2013). It is believed that linear F-actin polymerization driven by the ER-localized inverted formin 2 (INF2) (Korobova et al., 2013) and transient assembly of branched F-actin in the outer mitochondrial membrane lead by actin regulatory factors such as cortactin, cofilin and the Arp2/3 complexes (Li et al., 2015) provide the initial mitochondrial constriction that allows Drp1 to give the second and final cut to the mitochondria. Similarly, we observed here that actin polymerization inhibition with Latrunculin B and cytochalasin D prevents the fragmentation of the mitochondrial network induced by IP3R inhibition. Since SIRT1 was active and deacetylated cortactin promotes actin polymerization and branching (Zhang et al., 2009) we were particularly interested to determine the presence of this protein on the mitochondrial membrane, however, we were unable to detect it nor could we image actin polymerization using mApple-lifeact (data not shown) as demonstrated before (Li et al., 2015; Moore et al., 2016). As the assembly of F-actin on mitochondria is very transient (Li et al., 2015; Moore et al., 2016) and likely more visible in some cell models over others, it is possible that we missed it. Nevertheless, we clearly show that actin is involved and that the knockdown of cortactin prevents the mitochondrial fragmentation induced by IP3R inhibition. Although in most scenarios mitochondrial fragmentation is mediated by Drp1, at an early time point (1–4 h) after inhibition of Ca^2+^ transfer to the mitochondria, we observed mitochondrial fragmentation that proceeds without Drp1 activation. Similarly, infection with the human bacterial pathogen *Listeria monocytogenes* causes transient mitochondrial fragmentation (Stavru and Cossart, 2011) in a Drp1 independent fashion (Stavru et al., 2013). In this case, the bacterial toxin listeriolysin O (LLO) causes a significant drop in the ΔΨm, respiration and ATP levels probably as a result of overwhelming mitochondria with Ca^2+^ caused by influx from the extracellular space induced by the formation of ion permeable pores in the plasma membrane by LLO (Stavru et al., 2013). Although the above described changes have been observed in Drp1-dependent fragmentation (Pagliuso et al., 2018), why Drp1 is not recruited in this context is unknown. In addition, accumulating evidence suggests the presence of a Drp1-independent fission mechanism involved in the mitochondrial quality control (Soubannier et al., 2012; McLelland et al., 2014; Yamashita et al., 2016). For example, ROS induced protein damage triggers the generation of mitochondria-derived vesicles (MDVs), which bud off the mitochondria and are delivered to the lysosomes in a Parkin and Pink1 dependent fashion. Interestingly, and in agreement with our data, ΔΨm does not drop (Soubannier et al., 2012; McLelland et al., 2014). Also, using live cell imaging, Kanki’s group show that upon induction of mitophagy, mitochondria makes a bud that is wrapped and excised without the participation of Drp1 by an isolation membrane formed on the mitochondrial surface (Yamashita et al., 2016). Altogether, these works confirm the existence of Drp1-independent mitochondrial fragmentation mechanisms, and the mechanistic details await to be unveiled.

**FIGURE 7:**
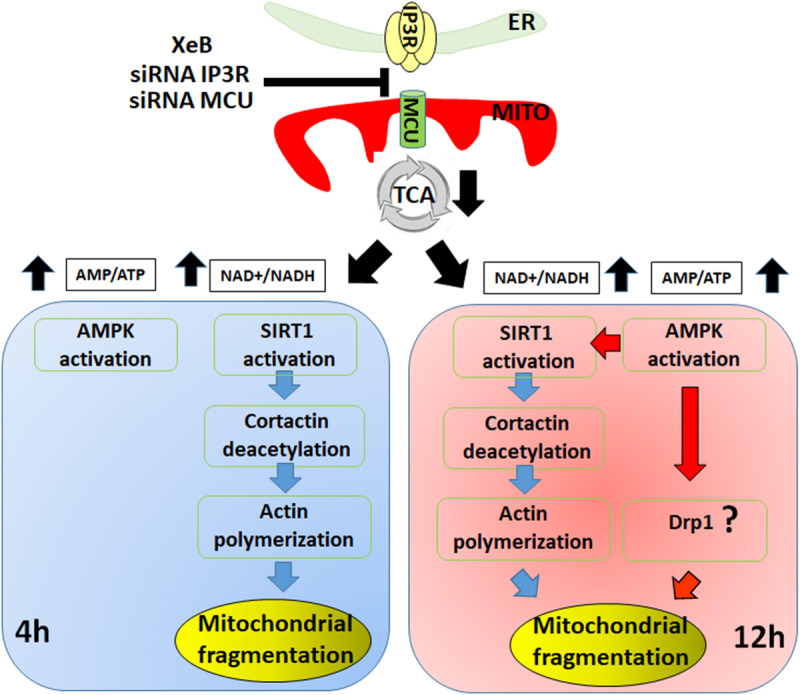
Different temporal effects of SIRT1 and AMPK on mitochondrial fragmentation. The initial fragmentation of the mitochondrial network induced by inhibition of the Ca^2+^ transfer to the mitochondria is mediated by SIRT1 deacetylation of cortactin and its effect on the actin polymerization (left panel). Upon prolonged inhibition of Ca^2+^ transfer to the mitochondria the mitochondrial fragmentation is sustained by AMPK activation, likely through Drp1 (right panel).

Here, we described a new mechanism of mitochondrial fragmentation induced by bioenergetic/metabolic stress that at an early time point proceeds in a Drp1-independent fashion, driven mainly by the actin cytoskeleton and the deacetylation of cortactin by SIRT1. At later time points, AMPK prevails and orchestrates the phenomenon (Figure 7). Further work is necessary to understand the exact temporal/spatial relationship between SIRT1 and AMPK in this phenomenon and whether the actin cytoskeleton itself accounts for the mitochondrial fragmentation or if it is the result of undescribed actin-associated proteins.

## Materials and Methods

### Reagents

#### Antibodies

From Cell Signaling Technology: Phospho-α1-AMPK (Thr172), α1-AMPK, IP3R-1, p53, acetyl-p53, cortactin, Drp1. From BD Laboratories: β-tubulin, IP3R-3. From Sigma: MCU, acetyl-cortactin. From Abcam: Tomm20. Secondary antibodies conjugated with peroxidase were purchased from GE Healthcare Life Science. TMRE, Fluo-4, mitotracker, BAPTA-AM, BCECF-AM, DAPI, and secondary antibodies conjugated with Alexa 488, 546, 633 were from Molecular Probes. *Chemicals*: methyl-pyruvate, FCCP, oligomycin, rotenone, nicotinamide (NMN), latrunculin B (LatB), cytochalasin D (CytD), and Hanks’ balanced salt solution were purchased from Sigma. EX527 and compound C were purchased from Tocris Bioscience. Xestospongin B was extracted and purified from the marine sponge *Xestospongia exigua* as described before (Jaimovich et al., 2005).

### Cell Culture and Transfection

HeLa, U-87, HEK293, primary human fibroblast (PHF), SIRT1-KO MEF cells (kindly provided by Dr. Guarente, Massachusetts Institute of Technology), Drp1-KO HCT116 cells and Mff-KO cells (kindly provided by Dr. Karbowski, University of Maryland) were maintained in DMEM media (GIBCO) supplemented with 10% (v/v) FBS. MCF10A were maintained in DMEM/F12 supplemented with 5% (v/v) horse serum, 20 ng/ml EGF, 0.5 mg/ml hydrocortisone, 100 ng/ml cholera toxin, 10 μg/ml insulin. All cells were grown at 37°C (95%/5% air/CO_2_) in the presence of 100 U ml^–1^ penicillin, 100 μg ml^–1^ streptomycin and 0.25 μg ml^–1^ fungizone (Gibco). To knockdown MCU we used a mix of two siRNA previously described (De Stefani et al., 2011):

5′- GCCAGAGACAGACAAUACUtt-3′
3′-ttCGGUCUCUGUCUGUUAUGA -5′ and
5′- GGGAAUUGACAGAGUUGCUtt-3′
3′-ttCCCUUAACUGUCUCAACGA -5′.

Type 1 and 3 IP3Rs were simultaneously silenced with a specific siRNA designed with the siRNA center tool from Dharmacon (GE Healthcare Dharmacon):

5′-GUGAGAAGCAGAAGAAGGAUU-3′
3′-UUCACUCUUCGUCUUCUUCCU-5′

The pLentiLox3.7(CMV)EGFP vector engineered to simultaneously produce cortactin shRNA and GFP (J Cell Sci. 2013 Jan 1;126(Pt 1):149-62. doi: 10.1242/jcs.110742) was kindly provided by Dr. Carvallo from Coimbra University, Portugal.

The pcDNA3-Drp1K38A was a gift from Alexander van der Bliek & Richard Youle (Addgene plasmid # 45161^1^; RRID:Addgene_45161) and the mCh-Drp1 was a gift from Gia Voeltz (Addgene plasmid # 49152^2^; RRID:Addgene_49152).

All transfections of siRNA were performed with a Nucleofector^®^ electroporator (Amaxa Biosystems).

### Immunofluorescence and Confocal Live Imaging

For Immunofluorescence, cells were fixed in 4% PFA (Electron microscopy sciences) for 15 min at room temperature, permeabilized with 0.1% Triton X-100, blocked in 5% BSA for 60 min, and incubated with primary TOMM20 antibody at 4°C overnight (Abcam #ab221292). Cells were washed with PBS/BSA and incubated with secondary antibody, Alexa-488 or Alexa-546 secondary antibody (Molecular Probes, #A-11008, #A-32732), at room temperature for 90 min and then mounted in Prolong gold antifade reagent with DAPI (Molecular Probes, #P36931). For live imaging cells were maintained in a Tokai Hit incubation chamber. Visual examination was performed on a Nikon A1R confocal microscope equipped with Perfect Focus using a 63X plan apo lens/NA 1.4. For quantification of mitochondrial morphology scoring was done blind to treatment. “Fragmented” was defined as cells in which the majority of mitochondria were spherical (no clear length/width or length below 1 μm), “medium” were cells with a majority of mitochondria between 1 and 4 μm and networked cells with a majority of mitochondria longer than 4 μm.

### Calcium Signaling

Imaging of spontaneous changes in cytoplasmic Ca^2+^ concentration in HeLa cells was accomplished by TIRF microscopy using a Nikon Eclipse Ti inverted TIRF microscope equipped with an apo TIRF 60x, 1.49 NA lens and stage-top Tokai Hit incubator. Cells were labeled with freshly prepared Fluo-4 (5 μM) and imaged a 37°C and 5% CO2 at a very shallow laser angle, imaging about 200 nm into the cell. The gain on the Andor iXon EMCCD camera was set to 300 (maximum) and images were collected every 50 ms for 2 min.

### Western Blotting and Treatments

Drugs were added in fresh media as indicated. Treatments were terminated by rapid removal of media with cells on ice, followed by cell lysis with Cytobuster protein extraction reagent (Novagen) supplemented with protease and phosphatase inhibitors (complete PhosSTOP, Roche). Protein extracts were separated in 4%, 10% or 15% SDS-polyacrylamide gels and transferred to PDVF membranes (Millipore). Blocking was at room temperature for 1 h in 5% fat-free milk, and membranes were incubated overnight at 4°C with primary antibody, and then for 1 h at room temperature with a secondary antibody conjugated to horseradish peroxidase. Chemiluminescence detection used ECL-plus reagent (Pierce) and a series of timed exposure images were acquired with a FluorChem Q system (ProteinSimple) to ensure densitometric analyses were performed at exposures within the linear range. To ensure equal protein loading across gels, membranes were submerged in stripping buffer (Restore Western blot stripping buffer; Pierce), incubated at 37°C for 20 min, and re-probed with a loading control antibody. Image J was used for densitometric analysis.

### Cellular Oxygen Consumption

Oxygen consumption rate (OCR) was measured at 37^*o*^C using an XF^*e*^96 extracellular analyzer (Seahorse Bioscience, North Billerica, MA, United States). 1.5 × 10^4^ cells per well were seeded onto CELL-TAK (BD Bioscience) pre-treated plates and allowed to attach for 24 h, and then were treated either with 5 μM XeB or the vehicle as a control and loaded into the machine in fresh un-buffered seahorse media. For the experiments with siRNA treated cells, after the transfected cells were seeded onto CELL-TAK (BD Bioscience) pre-treated plates. On the seahorse, cells were sequentially exposed to 1 μM oligomycin, 300 nM carbonylcyanide p-trifluoromethoxyphenylhydrazone (FCCP) and 100 nM rotenone. Basal and maximal OCR were calculated as previously described (cardenas 2010). Data were normalized for protein concentration by lysing samples after each experiment.

### NAD+/NADH Measurements

The NAD+/NADH ratio was measured using a commercial kit (BioVision, K337-100) according to the manufacturer’s instructions. Briefly, cells were incubated either with 5 μM XeB or vehicle for 1 or 4 h. Cells were then washed twice with PBS, scraped off the dishes and pelleted. NAD+ and NADH were extracted and the samples were subjected to two freeze thaw cycles and centrifuged. Aliquots of each sample were heated at 60°C for 30 min to decompose NAD+. Samples were then loaded into 96-well plates for absorbance measurements at 450 nm.

### AMP/ATP Measurements

Nucleotides were extracted from the cells using perchloric acid, neutralized, and frozen for subsequent HPLC analysis (Kochanowski et al., 2006). AMP, ADP, and ATP in extracted samples were quantified using ion-pair reverse-phase HPLC, with a C18 RP column, under isocratic elution conditions in 200 mM phosphate, 5 mM tetrabutylammonium phosphate, and 3% acetonitrile.

### Electron Microscopy

Cells were fixed in 2.5% glutaraldehyde in 0.1M cacodylate buffer, postfixed in 2% osmium tetroxide, dehydrated and embedded in Epon, and examined with a high-voltage electron microscope (Philips EM 410).

### Analysis and Statistics

All data are summarized as mean ± SEM; significance of differences was assessed using unpaired *t*-tests. Differences were accepted as significant at the 95% level (p < 0.05).

## Data Availability Statement

All datasets generated for this study are included in the article/Supplementary Material.

## Author Contributions

AL and CC directed and elaborated the design of the study, interpreted and analyzed the data, and wrote the manuscript. AL, UA-C, PF, and GB performed the research and analyzed the data. JM participated in the design of the study and facilitated the Xestosopongin B. CG-B participate in the design and generation of cortactin data. All authors read and approved the final manuscript version.

## Conflict of Interest

The authors declare that the research was conducted in the absence of any commercial or financial relationships that could be construed as a potential conflict of interest.

## Footnotes

1 http://n2t.net/addgene:45161

2 http://n2t.net/addgene:49152

## Supplementary Material

FIGURE S1 Electron microscopy reveals that the inhibition of IP3R-mediated calcium transfer to the mitochondria causes mitochondrial fragmentation without affecting the mitochondrial inner structure. HeLa cells control and treated with 5 μM XeB for 4 h were visualized by electron microscopy at low magnification (6000×, bar = 2 μm) **(A)** or high magnification (43000×, bar = 5 nm) **(B)**.

FIGURE S2 Inhibition of IP3R-mediated calcium transfer to the mitochondria causes mitochondrial fragmentation in several cell lines. Representative confocal images of glioblastoma U87 cell line **(A)**, breast MCF10A cell line **(B)**, primary human fibroblast (PHF) **(C)**, and HEK293 cell line **(D)** treated with 5 μM XeB for 4 h and then immunostained with anti-TOMM20 to detect mitochondria. Bar: 10 μm. **(E)** Mitochondrial morphology analysis of U87, MCF10A, PHF, and HEK293 cell lines treated with 5 μM XeB for 4 h; fragmented (black), ≤1 μm, medium (light gray), ≥1 and ≤4 μm, networked (dark gray), ≥4 μm. Data represent mean ± SEM of 3 independent experiments. In each experiment 150 cells/condition were scored. ^∗∗∗^*p* < 0.001.

FIGURE S3 Calcium chelation induces mitochondrial fragmentation. **(A)** Representative confocal images of HeLa cells loaded with the calcium chelator 1 μM BAPTA-AM or the pH indicator 1 μM BCECF-AM as control for 1 h. **(B)** Mitochondrial morphology analysis of HeLa cells loaded with 1 μM BAPTA-AM or 1 μM BCECF-AM as control for 1 h; fragmented (black), ≤1 μm, medium (light gray), ≥1 and ≤4 μm, networked (dark gray), ≥4 μm. Data represent means ± SEM of 3 independent experiments. In each experiment 150 cells/condition were scored. **(C)** Hela cells were labeled with the mitochondrial membrane potential (ΔΨm) dye TMRE in non-quenching mode (8 nM TMRE for 1 h), treated with 5 μM XeB and imaged every 15 min. Bar = 10 μm. **(D)** Hela cells were labeled with TMRE, treated with 5 μM XeB or FCCP and changes in the mitochondrial membrane potential (ΔΨm) determined by cytometry. ^∗∗^*p* < 0.01, ^∗∗∗^*p* < 0.001. **(E)** Hela cells were treated or not with 5 μM XeB for 4 h and then immunostained with anti-TOMM20 (red) and LC3 (green) to detect mitochondria and autophagosomes, respectively. Bar: 10 μm.

FIGURE S4 Activation of AMPK upon inhibition of IP3R. **(A)** AMP/ATP ratio increases significantly 1 and 4 h after treatment with 5 uM XeB. **(B)** Representative Western blot of AMPK phosphorylation on threonine-172 (P-AMPK) in HeLa cells treated (CT) with 5 μM XeB or with vehicle for 4 h. Bar graph: P-AMPK/AMPK expressed as average fold increase over basal levels (control cells, CT). Mean ± SEM of 3 independent experiments with 4 replicates each. ^∗∗^*P* < 0.01 compared to control. **(C)** Representative confocal images of HeLa cells labeled with 8 nM TMRE to visualize mitochondria treated simultaneously with 5 μM XeB and compound C (CC) for 4 h. Bar: 10 μm. **(D)** Mitochondrial morphology analysis of HeLa cells treated simultaneously with 5 μM XeB and compound C (CC, 10 μM) for 4 h **(C)**; fragmented (black), ≤1 μm, medium (light gray), ≥1, and ≤4 μm, networked (dark gray), ≥4 μm. Data represent mean ± SEM of 3 independent experiments. In each experiment 150 cells/condition were scored. ^∗∗^*p* < 0.01, ^∗∗∗^*p* < 0.001. ns, not significant.

FIGURE S5 Activation of SIRT1 with nicotinamide induces mitochondrial fragmentation. **(A)** Representative Western blot of SIRT1 in cells treated with 5 μM XeB for 1 h. Bar graph: SIRT1/tubulin expressed as average fold increase over basal levels (control cells, CT). Mean ± SEM of 3 independent experiments with 3 replicates each. **(B)** Representative confocal images of HeLa cells labeled with 8 nM TMRE to visualize mitochondria treated with 1 mM β-nicotinamide mononucleotide (NMN) for 12 h. Bar: 10 μm. **(C)** Mitochondrial morphology analysis of HeLa cells treated with 1 mM NMN for 12 h; fragmented (black), ≤1 μm, medium (light gray), ≥1 and ≤4 μm, networked (dark gray), ≥4 μm. Data represent mean ± SEM of 3 independent experiments. In each experiment 150 cells/condition were scored. ^∗∗∗^*p* < 0.001.

FIGURE S6 The presence of a Drp1 dominant negative does not prevent mitochondrial fragmentation induced by IP3R Inhibition. **(A)** Representative confocal images of the same HeLa cells transfected with Drp1-mCherry and labeled with mitotracker far-red treated with 5 μM XeB or vehicle for 4 h. Arrows point to Drp1-mCherry puncta in the mitochondria. Bar: 10 μm. **(B)** Representative Western blot of HeLa cells transfected with a Drp1-dominant negative (K38A) or Mock as control. Bar graph: Drp1/tubulin expressed as average fold increase over basal levels (control cells, Mock). Mean ± SEM of 3 independent experiments with 3 replicates each. ^∗∗∗^*P* < 0.001 compared to control. **(C)** Representative confocal images of HeLa cells transfected with a Drp1-dominant negative (K38A) treated or not with 5 μM XeB for 4 h and then immunostained with anti-TOMM20 to detect mitochondria. Bar: 10 μm. **(D)** Mitochondrial morphology analysis of HeLa cells transfected with a Drp1-dominant negative (K38A) treated or not with 5 μM XeB for 4 h **(C)**; fragmented (black), ≤1 μm, medium (light gray), ≥1 and ≤4 μm, networked (dark gray), ≥4 μm. Data represent means ± SEM of 3 independent experiments. In each experiment 150 cells/condition were scored. ^∗∗^*p* < 0.01, ^∗∗∗^*p* < 0.001. ns, not significant.

FIGURE S7 Cortactin and the polymerization of the actin cytoskeleton are required for the mitochondrial fragmentation induced by inhibition of the Ca^2+^ transfer to the mitochondria. **(A)** Representative confocal images of HeLa cells treated simultaneously with 5 μM XeB and 500 nM latrunculin B for 4 h. Mitochondria were labeled with TOMM20 (magenta) and the actin cytoskeleton was labeled with phalloidin (green). Bar: 10 μm. **(B)** Mitochondrial morphology analysis of HeLa cells treated simultaneously with 5 μM XeB and 500 nM latrunculin B for 4 h **(A)**; Fragmented (black), ≤1 μm, medium (light gray), ≥1 and ≤4 μm, networked (dark gray), ≥4 μm. Data represent mean ± SEM of 3 independent experiments. In each experiment 150 cells/condition were scored. ^∗∗^*p* < 0.01, ^∗∗∗^*p* < 0.001. ns, not significant. **(C)**. Representative confocal images of HeLa cells transfected with a vector engineered to simultaneously produce cortactin shRNA and GFP, loaded with mitotracker to visualize mitochondria and treated with 5 μM XeB for 4 h. Digital zoom of the square areas in the middle panel are shown in the right panel. Bar: 10 μm.

FIGURE S8 Overnight inhibition of AMPK protects the mitochondrial network from the fragmentation induced by inhibition of the Ca^2+^ transfer to the mitochondria. **(A)** Hela cells treated with 5 μM XeB for several hours were labeled with propidium iodide (PI) and cell death determined by flow cytometry. **(B)** Hela cells treated with high concentration of XeB (7.5 and 10 μM) for 12 h were labeled with propidium iodide (PI) and cell death determined by flow cytometry. **(C)** Confocal images of Hela cells labeled with 8 nM TMRE to visualize mitochondria simultaneously treated with 5 μM XeB for 12 h and either the AMPK inhibitor compound C (CC) or the SIRT1 inhibitor EX527 (12.5 μM). Bar: 10 μm. **(D)** Mitochondrial morphology analysis of HeLa cells simultaneously treated with 5 μM XeB for 12 h and either the AMPK inhibitor compound C (CC) or the SIRT1 inhibitor EX527 (12.5 μM); fragmented (black), ≤1 μm, medium (light gray), ≥1 and ≤4 μm, networked (dark gray), ≥4 μm. Data represent mean ± SEM of 3 independent experiments. In each experiment 150 cells/condition were scored. ^∗^*p* < 0.05, ^∗∗∗^*p* < 0.001. ns, not significant.

FIGURE S9 The absence of SIRT1 partially protects the mitochondrial network after an overnight inhibition of calcium transfer to the mitochondria. **(A)** Representative confocal images of MEF SIRT1 knockout (KO) or WT cells labeled with 8 nM TMRE to visualize mitochondria treated with 5 μM XeB for 12 h. (1Bar: 10 μm). **(D)** Mitochondrial morphology analysis of MEF SIRT1 knockout (KO) or WT cells treated with 5 μM XeB for 12 h.; fragmented (black), ≤1 μm, medium (light gray), ≥1 and ≤4 μm, networked (dark gray), ≥4 μm. Data represent mean ± SEM of 3 independent experiments. In each experiment 150 cells/condition were scored. ^∗∗^*p* < 0.001, ^∗∗∗^*p* < 0.001.
