# Supplementary figures and images for: Concerted Action of AMPK and Sirtuin-1 Induces Mitochondrial Fragmentation Upon Inhibition of Ca2+ Transfer to Mitochondria
Source: Front Cell Dev Biol. 2020 May 25;8:378. doi: 10.3389/fcell.2020.00378 (PMC7261923; doi:10.3389/fcell.2020.00378)

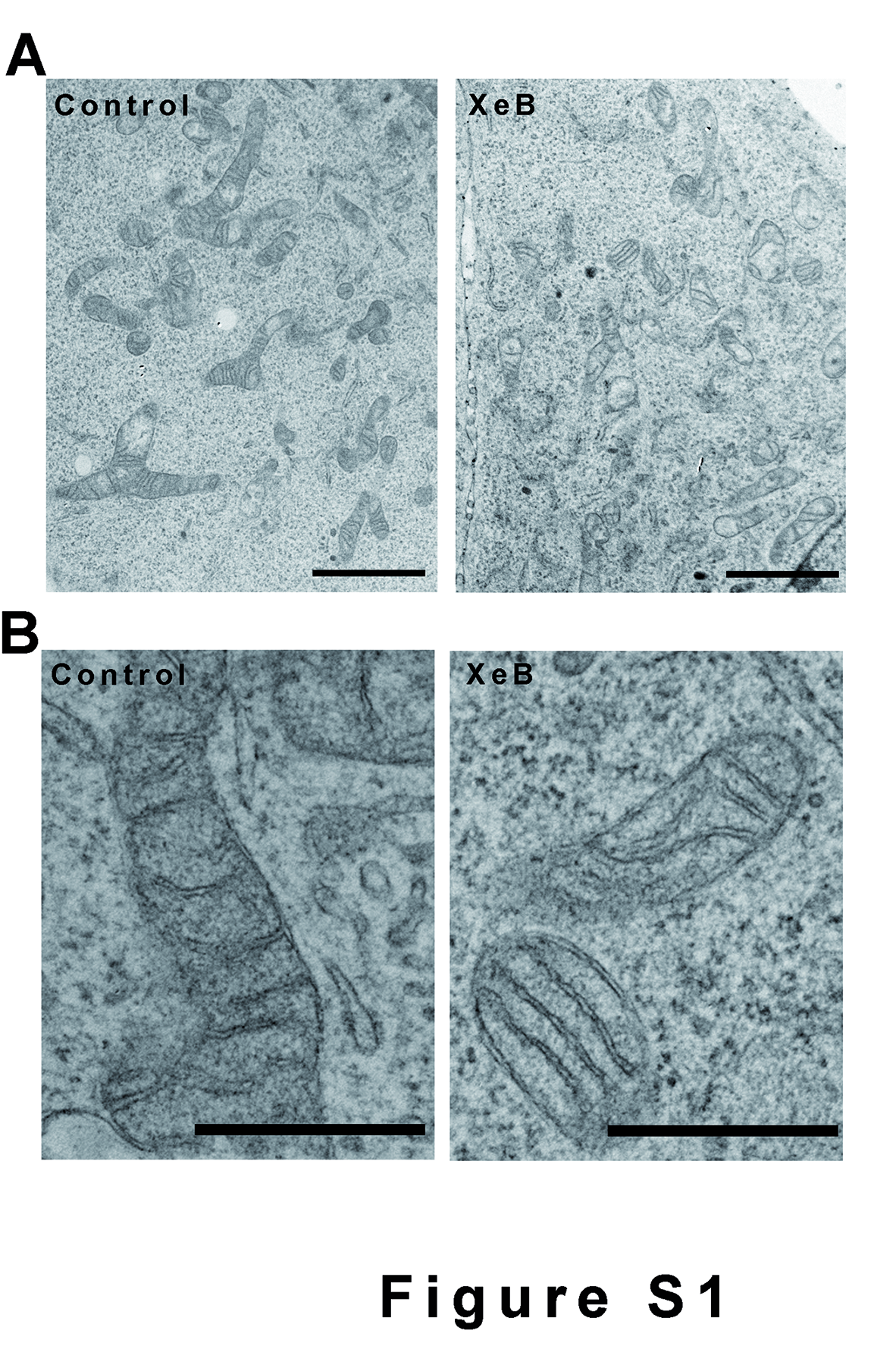

Supplement: FIGURE S1 — Electron microscopy reveals that the inhibition of IP3R-mediated calcium transfer to the mitochondria causes mitochondrial fragmentation without affecting the mitochondrial inner structure. HeLa cells control and treated with 5 μM XeB for 4 h were visualized by electron microscopy at low magnification (6000×, bar = 2 μm) (A) or high magnification (43000×, bar = 5 nm) (B). [file Image_1.tif]

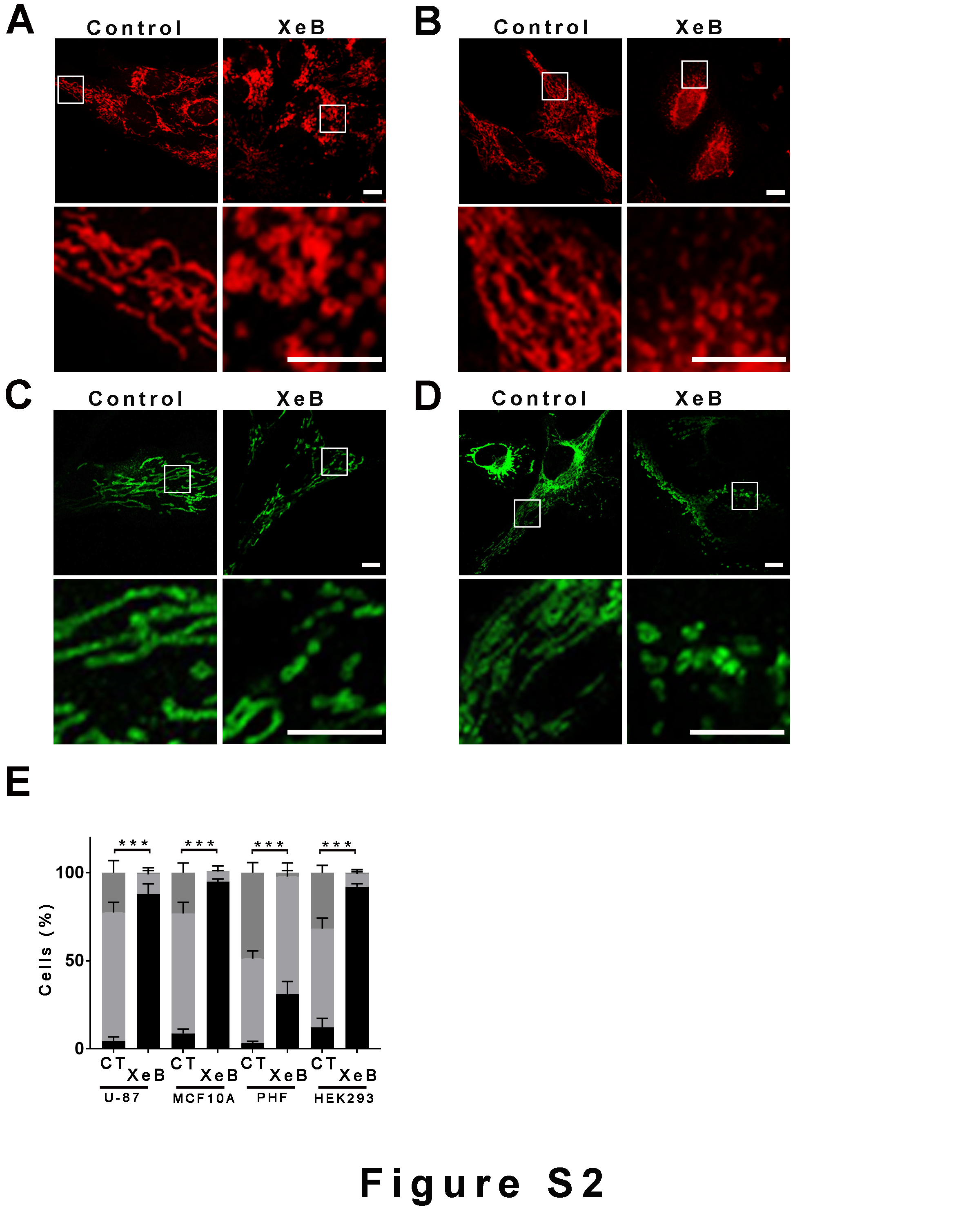

Supplement: FIGURE S2 — Inhibition of IP3R-mediated calcium transfer to the mitochondria causes mitochondrial fragmentation in several cell lines. Representative confocal images of glioblastoma U87 cell line (A), breast MCF10A cell line (B), primary human fibroblast (PHF) (C), and HEK293 cell line (D) treated with 5 μM XeB for 4 h and then immunostained with anti-TOMM20 to detect mitochondria. Bar: 10 μm. (E) Mitochondrial morphology analysis of U87, MCF10A, PHF, and HEK293 cell lines treated with 5 μM XeB for 4 h; fragmented (black), ≤1 μm, medium (light gray), ≥1 and ≤4 μm, networked (dark gray), ≥4 μm. Data represent mean ± SEM of 3 independent experiments. In each experiment 150 cells/condition were scored. ∗∗∗p < 0.001. [file Image_2.tif]

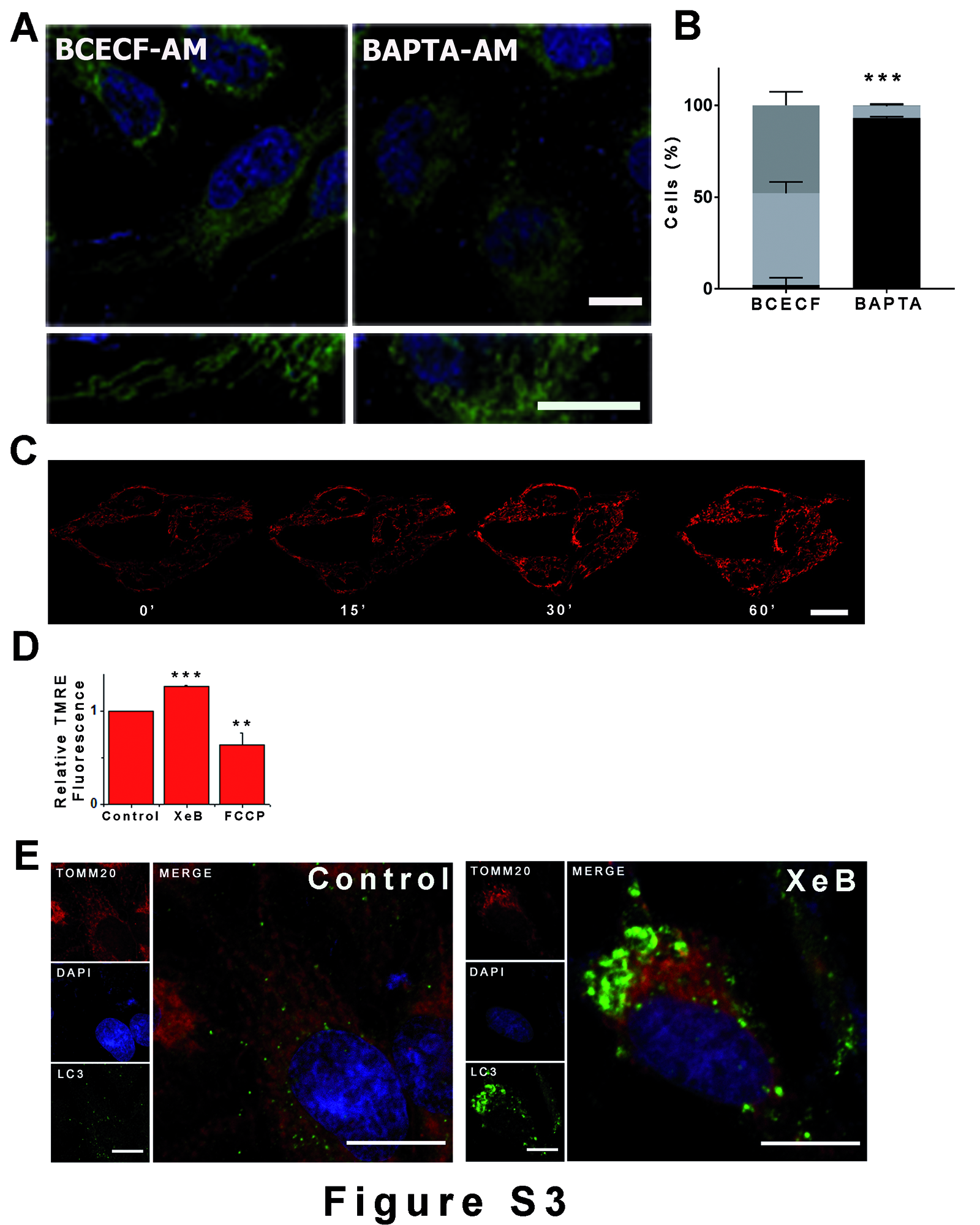

Supplement: FIGURE S3 — Calcium chelation induces mitochondrial fragmentation. (A) Representative confocal images of HeLa cells loaded with the calcium chelator 1 μM BAPTA-AM or the pH indicator 1 μM BCECF-AM as control for 1 h. (B) Mitochondrial morphology analysis of HeLa cells loaded with 1 μM BAPTA-AM or 1 μM BCECF-AM as control for 1 h; fragmented (black), ≤1 μm, medium (light gray), ≥1 and ≤4 μm, networked (dark gray), ≥4 μm. Data represent means ± SEM of 3 independent experiments. In each experiment 150 cells/condition were scored. (C) Hela cells were labeled with the mitochondrial membrane potential (ΔΨm) dye TMRE in non-quenching mode (8 nM TMRE for 1 h), treated with 5 μM XeB and imaged every 15 min. Bar = 10 μm. (D) Hela cells were labeled with TMRE, treated with 5 μM XeB or FCCP and changes in the mitochondrial membrane potential (ΔΨm) determined by cytometry. ∗∗p < 0.01, ∗∗∗p < 0.001. (E) Hela cells were treated or not with 5 μM XeB for 4 h and then immunostained with anti-TOMM20 (red) and LC3 (green) to detect mitochondria and autophagosomes, respectively. Bar: 10 μm. [file Image_3.tif]

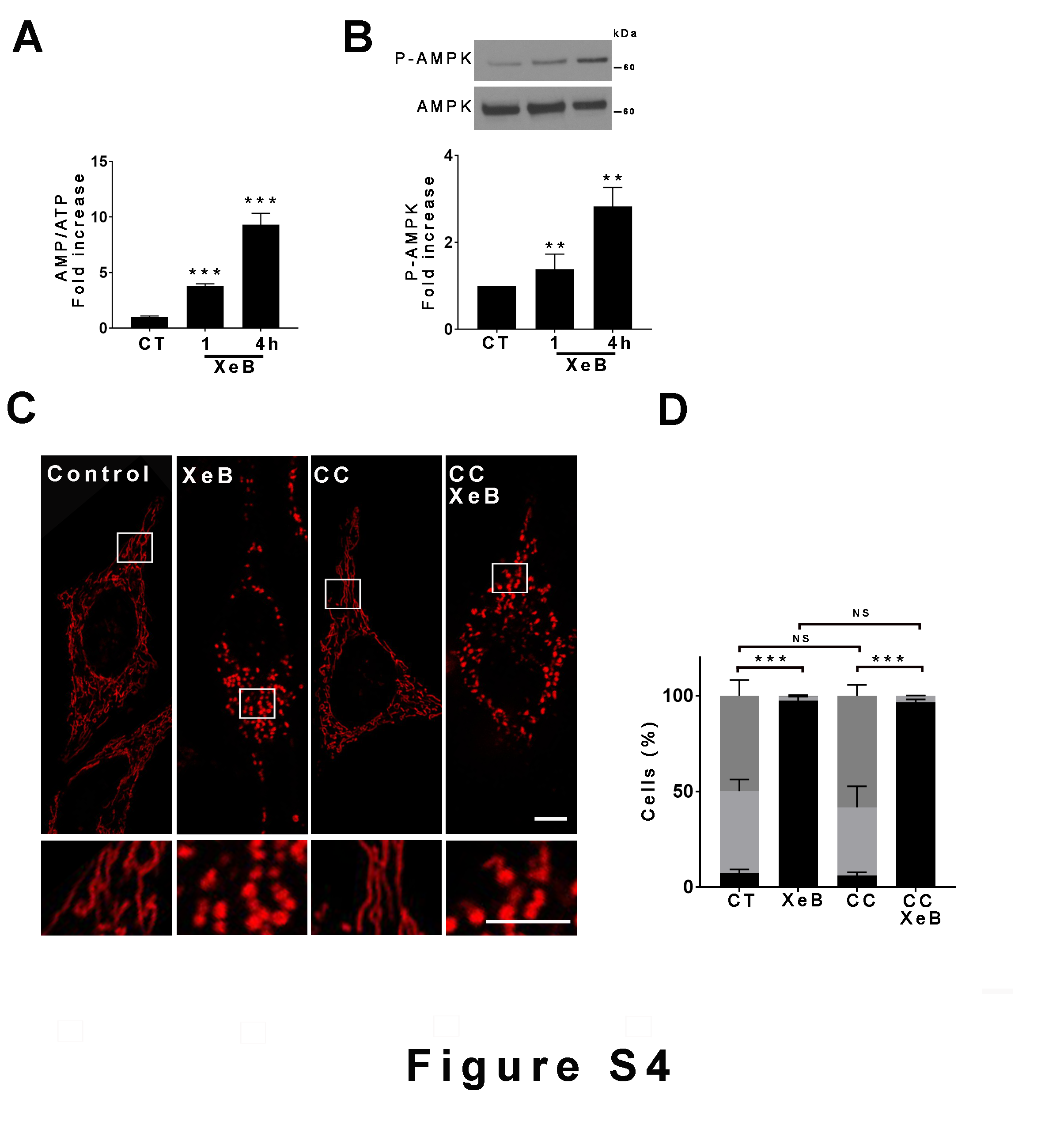

Supplement: FIGURE S4 — Activation of AMPK upon inhibition of IP3R. (A) AMP/ATP ratio increases significantly 1 and 4 h after treatment with 5 uM XeB. (B) Representative Western blot of AMPK phosphorylation on threonine-172 (P-AMPK) in HeLa cells treated (CT) with 5 μM XeB or with vehicle for 4 h. Bar graph: P-AMPK/AMPK expressed as average fold increase over basal levels (control cells, CT). Mean ± SEM of 3 independent experiments with 4 replicates each. ∗∗P < 0.01 compared to control. (C) Representative confocal images of HeLa cells labeled with 8 nM TMRE to visualize mitochondria treated simultaneously with 5 μM XeB and compound C (CC) for 4 h. Bar: 10 μm. (D) Mitochondrial morphology analysis of HeLa cells treated simultaneously with 5 μM XeB and compound C (CC, 10 μM) for 4 h (C); fragmented (black), ≤1 μm, medium (light gray), ≥1, and ≤4 μm, networked (dark gray), ≥4 μm. Data represent mean ± SEM of 3 independent experiments. In each experiment 150 cells/condition were scored. ∗∗p < 0.01, ∗∗∗p < 0.001. ns, not significant. [file Image_4.tif]

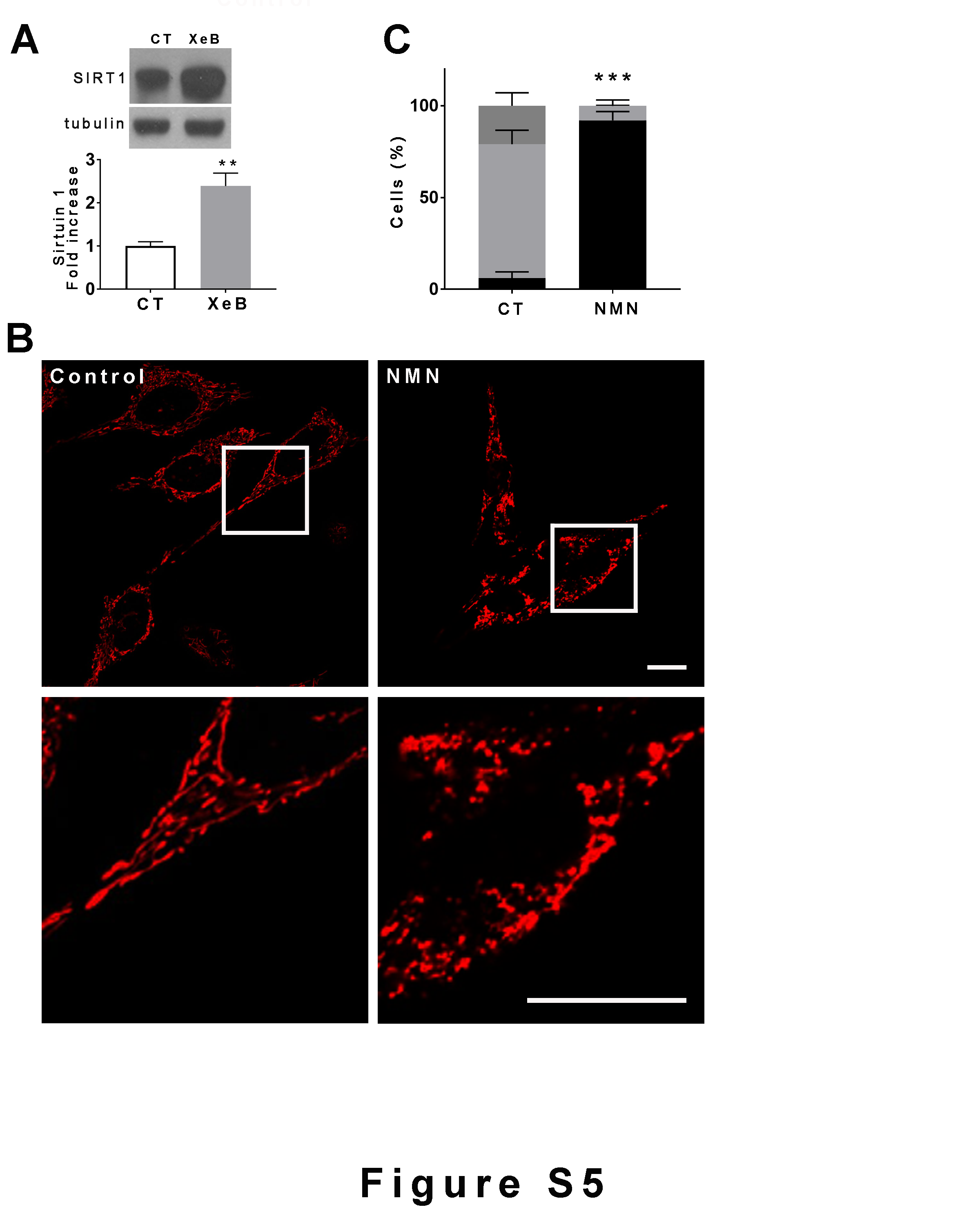

Supplement: FIGURE S5 — Activation of SIRT1 with nicotinamide induces mitochondrial fragmentation. (A) Representative Western blot of SIRT1 in cells treated with 5 μM XeB for 1 h. Bar graph: SIRT1/tubulin expressed as average fold increase over basal levels (control cells, CT). Mean ± SEM of 3 independent experiments with 3 replicates each. (B) Representative confocal images of HeLa cells labeled with 8 nM TMRE to visualize mitochondria treated with 1 mM β-nicotinamide mononucleotide (NMN) for 12 h. Bar: 10 μm. (C) Mitochondrial morphology analysis of HeLa cells treated with 1 mM NMN for 12 h; fragmented (black), ≤1 μm, medium (light gray), ≥1 and ≤4 μm, networked (dark gray), ≥4 μm. Data represent mean ± SEM of 3 independent experiments. In each experiment 150 cells/condition were scored. ∗∗∗p < 0.001. [file Image_5.tif]

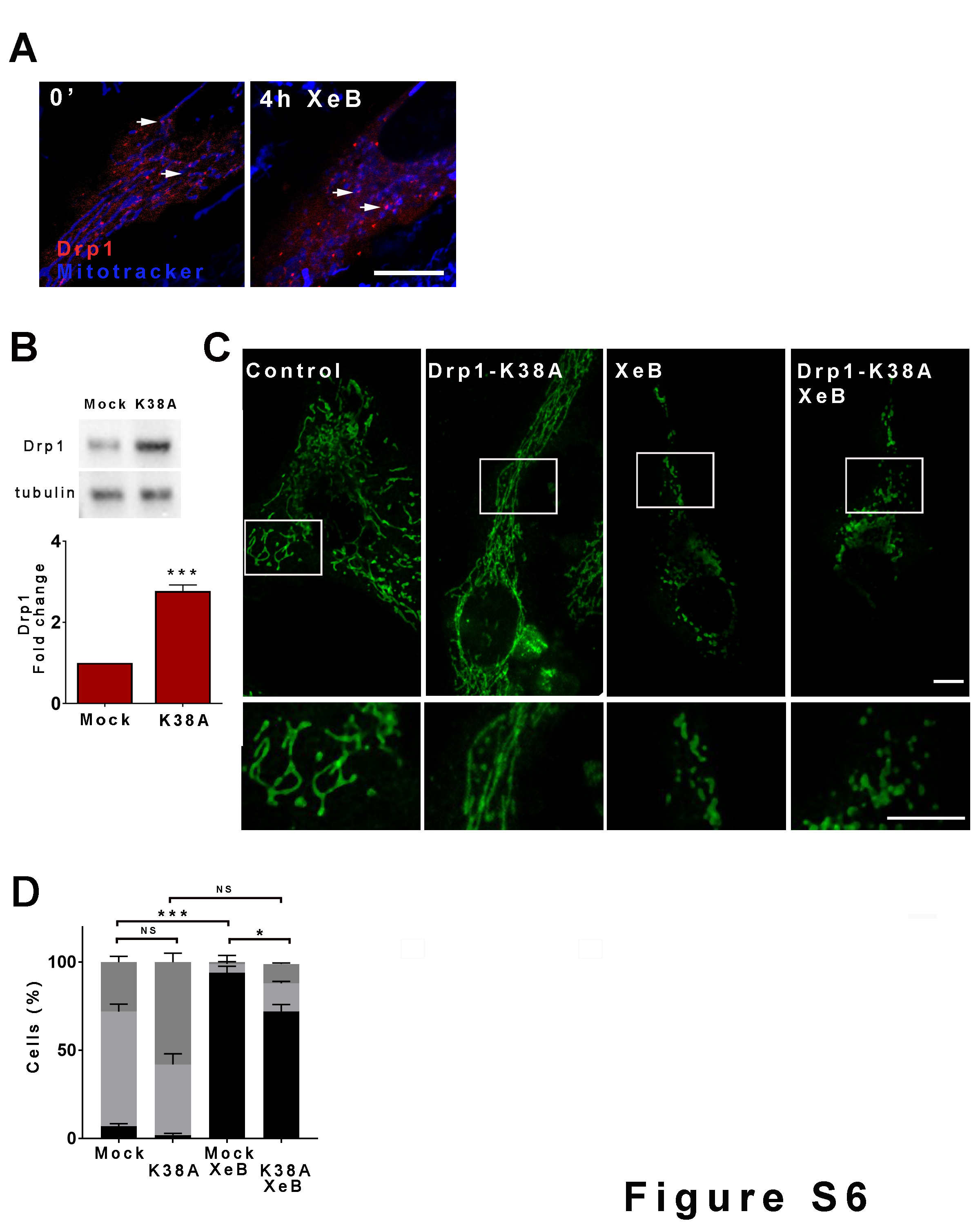

Supplement: FIGURE S6 — The presence of a Drp1 dominant negative does not prevent mitochondrial fragmentation induced by IP3R Inhibition. (A) Representative confocal images of the same HeLa cells transfected with Drp1-mCherry and labeled with mitotracker far-red treated with 5 μM XeB or vehicle for 4 h. Arrows point to Drp1-mCherry puncta in the mitochondria. Bar: 10 μm. (B) Representative Western blot of HeLa cells transfected with a Drp1-dominant negative (K38A) or Mock as control. Bar graph: Drp1/tubulin expressed as average fold increase over basal levels (control cells, Mock). Mean ± SEM of 3 independent experiments with 3 replicates each. ∗∗∗P < 0.001 compared to control. (C) Representative confocal images of HeLa cells transfected with a Drp1-dominant negative (K38A) treated or not with 5 μM XeB for 4 h and then immunostained with anti-TOMM20 to detect mitochondria. Bar: 10 μm. (D) Mitochondrial morphology analysis of HeLa cells transfected with a Drp1-dominant negative (K38A) treated or not with 5 μM XeB for 4 h (C); fragmented (black), ≤1 μm, medium (light gray), ≥1 and ≤4 μm, networked (dark gray), ≥4 μm. Data represent means ± SEM of 3 independent experiments. In each experiment 150 cells/condition were scored. ∗∗p < 0.01, ∗∗∗p < 0.001. ns, not significant. [file Image_6.tif]

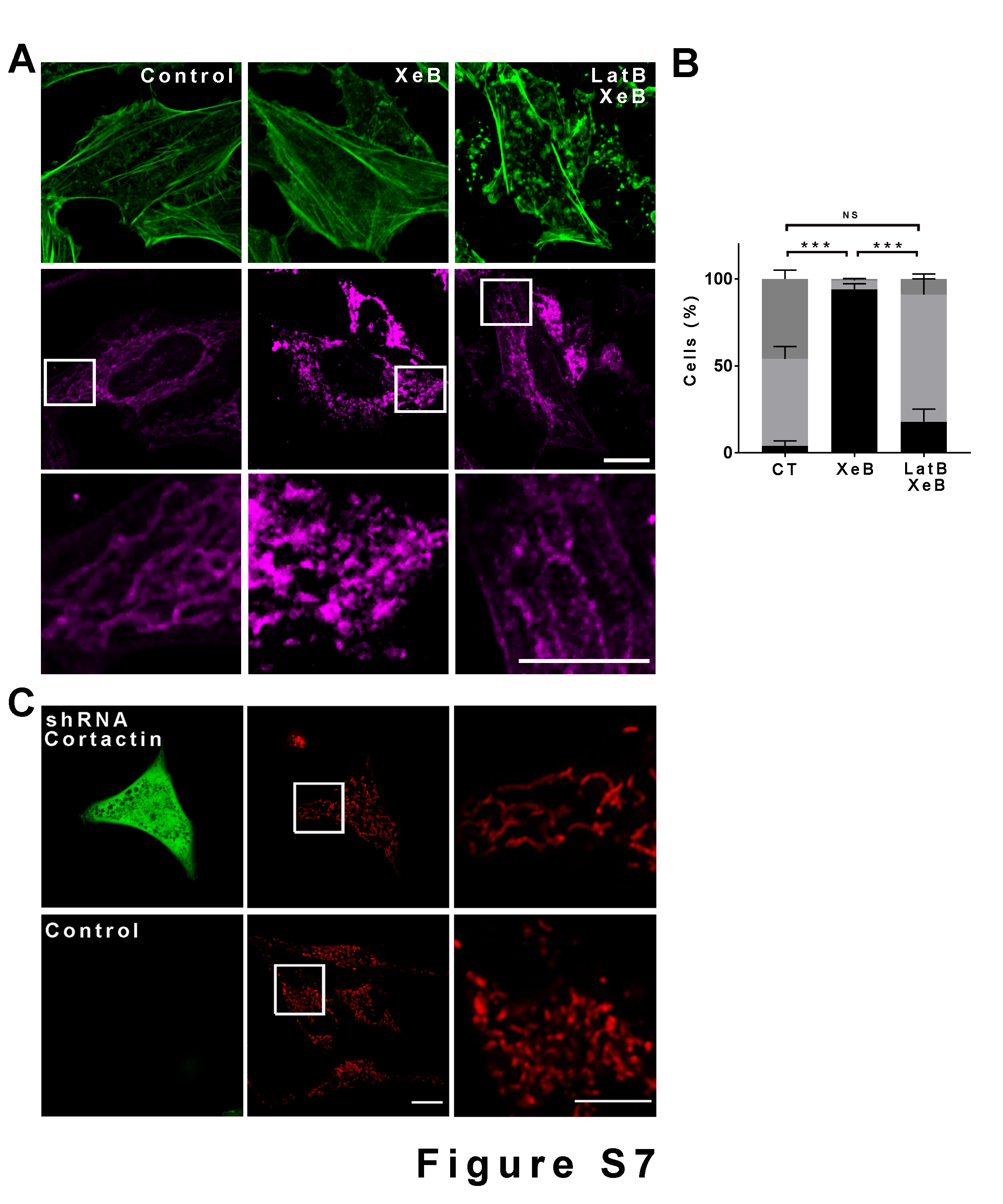

Supplement: FIGURE S7 — Cortactin and the polymerization of the actin cytoskeleton are required for the mitochondrial fragmentation induced by inhibition of the Ca2+ transfer to the mitochondria. (A) Representative confocal images of HeLa cells treated simultaneously with 5 μM XeB and 500 nM latrunculin B for 4 h. Mitochondria were labeled with TOMM20 (magenta) and the actin cytoskeleton was labeled with phalloidin (green). Bar: 10 μm. (B) Mitochondrial morphology analysis of HeLa cells treated simultaneously with 5 μM XeB and 500 nM latrunculin B for 4 h (A); Fragmented (black), ≤1 μm, medium (light gray), ≥1 and ≤4 μm, networked (dark gray), ≥4 μm. Data represent mean ± SEM of 3 independent experiments. In each experiment 150 cells/condition were scored. ∗∗p < 0.01, ∗∗∗p < 0.001. ns, not significant. (C). Representative confocal images of HeLa cells transfected with a vector engineered to simultaneously produce cortactin shRNA and GFP, loaded with mitotracker to visualize mitochondria and treated with 5 μM XeB for 4 h. Digital zoom of the square areas in the middle panel are shown in the right panel. Bar: 10 μm. [file Image_7.tif]

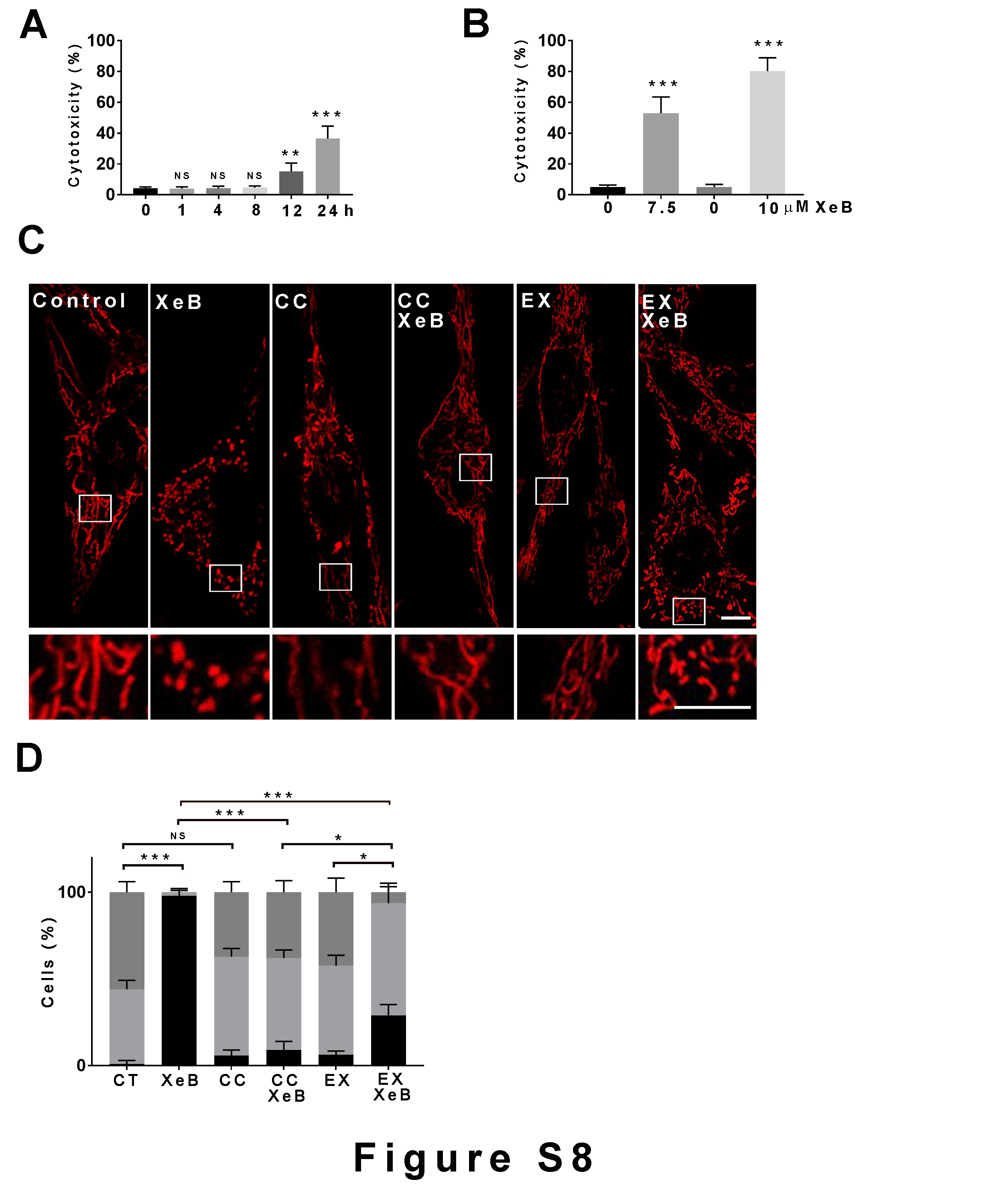

Supplement: FIGURE S8 — Overnight inhibition of AMPK protects the mitochondrial network from the fragmentation induced by inhibition of the Ca2+ transfer to the mitochondria. (A) Hela cells treated with 5 μM XeB for several hours were labeled with propidium iodide (PI) and cell death determined by flow cytometry. (B) Hela cells treated with high concentration of XeB (7.5 and 10 μM) for 12 h were labeled with propidium iodide (PI) and cell death determined by flow cytometry. (C) Confocal images of Hela cells labeled with 8 nM TMRE to visualize mitochondria simultaneously treated with 5 μM XeB for 12 h and either the AMPK inhibitor compound C (CC) or the SIRT1 inhibitor EX527 (12.5 μM). Bar: 10 μm. (D) Mitochondrial morphology analysis of HeLa cells simultaneously treated with 5 μM XeB for 12 h and either the AMPK inhibitor compound C (CC) or the SIRT1 inhibitor EX527 (12.5 μM); fragmented (black), ≤1 μm, medium (light gray), ≥1 and ≤4 μm, networked (dark gray), ≥4 μm. Data represent mean ± SEM of 3 independent experiments. In each experiment 150 cells/condition were scored. ∗p < 0.05, ∗∗∗p < 0.001. ns, not significant. [file Image_8.tif]

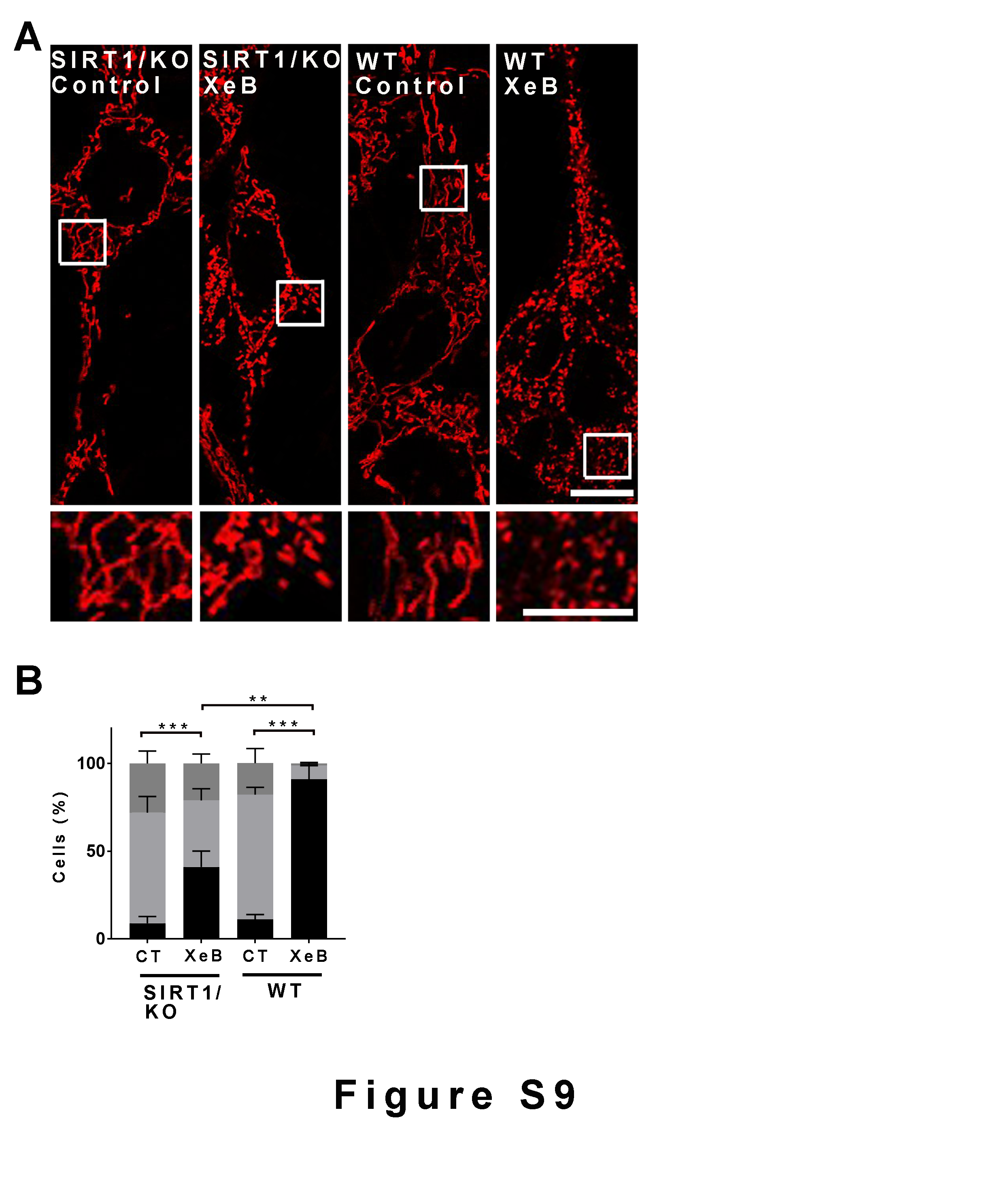

Supplement: FIGURE S9 — The absence of SIRT1 partially protects the mitochondrial network after an overnight inhibition of calcium transfer to the mitochondria. (A) Representative confocal images of MEF SIRT1 knockout (KO) or WT cells labeled with 8 nM TMRE to visualize mitochondria treated with 5 μM XeB for 12 h. (1Bar: 10 μm). (D) Mitochondrial morphology analysis of MEF SIRT1 knockout (KO) or WT cells treated with 5 μM XeB for 12 h.; fragmented (black), ≤1 μm, medium (light gray), ≥1 and ≤4 μm, networked (dark gray), ≥4 μm. Data represent mean ± SEM of 3 independent experiments. In each experiment 150 cells/condition were scored. ∗∗p < 0.001, ∗∗∗p < 0.001. [file Image_9.tif]
